# KIF13B establishes a CAV1-enriched microdomain at the ciliary transition zone to promote Sonic hedgehog signalling

**DOI:** 10.1038/ncomms14177

**Published:** 2017-01-30

**Authors:** Kenneth B. Schou, Johanne B. Mogensen, Stine K. Morthorst, Brian S. Nielsen, Aiste Aleliunaite, Andrea Serra-Marques, Nicoline Fürstenberg, Sophie Saunier, Albane A. Bizet, Iben R. Veland, Anna Akhmanova, Søren T. Christensen, Lotte B. Pedersen

**Affiliations:** 1 Department of Biology, Section of Cell Biology and Physiology, The August Krogh Building, University of Copenhagen, Universitetsparken 13, Copenhagen OE DK-2100, Denmark; 2 Cell Biology, Department of Biology, Faculty of Science, Utrecht University, Padualaan 8, CH Utrecht 3584, The Netherlands; 3 Inserm UMR-1163, Laboratory of Hereditary Kidney diseases, Paris 75015, France; 4 Paris Descartes Sorbonne Paris Cité University, Imagine Institut, Paris 75015, France

## Abstract

Ciliary membrane composition is controlled by transition zone (TZ) proteins such as RPGRIP1, RPGRIPL and NPHP4, which are vital for balanced coordination of diverse signalling systems like the Sonic hedgehog (Shh) pathway. Activation of this pathway involves Shh-induced ciliary accumulation of Smoothened (SMO), which is disrupted by disease-causing mutations in TZ components. Here we identify kinesin-3 motor protein KIF13B as a novel member of the RPGRIP1N-C2 domain-containing protein family and show that KIF13B regulates TZ membrane composition and ciliary SMO accumulation. KIF13B is upregulated during ciliogenesis and is recruited to the ciliary base by NPHP4, which binds to two distinct sites in the KIF13B tail region, including an RPGRIP1N-C2 domain. KIF13B and NPHP4 are both essential for establishment of a CAV1 membrane microdomain at the TZ, which in turn is required for Shh-induced ciliary SMO accumulation. Thus KIF13B is a novel regulator of ciliary TZ configuration, membrane composition and Shh signalling.

## Abstract (web summary)

The ciliary transition zone (TZ) regulates the protein and membrane composition of the primary cilium. Here the authors identify the kinesin-3 motor protein KIF13B as a regulator of TZ membrane composition that controls the ciliary accumulation of Smoothened, which is involved in activation of Sonic hedgehog signalling.

Primary cilia are microtubule-based sensory organelles that project from the surface of most non-dividing cells in our body and play pivotal roles in coordinating many different signalling pathways that regulate development, sensory perception and homeostasis^1^. Signalling pathways coordinated by primary cilia include Sonic hedgehog (Shh) (ref. 2), Wingless/Int (WNT) signalling^3^ and signalling via receptor tyrosine kinases^4^. Importantly, these pathways crosstalk extensively, and mutations in ciliary genes therefore impair multiple signalling pathways leading to diseases—ciliopathies—which are highly pleiotropic and may affect nearly all types of tissues and organs during development and in adulthood^5^.

Cilia consist of a microtubule axoneme that extends from a modified centriole called basal body and is surrounded by a bilayered lipid membrane. In many cell types, the proximal part of the cilium is embedded within a membrane invagination known as the ciliary pocket, which is a hotspot for exo- and endocytosis of vesicles destined to or derived from the ciliary membrane. The ciliary pocket membrane is also called the periciliary membrane, which demarcates the region between the plasma and ciliary membranes^6^,^7^. Although the ciliary membrane is continuous with that of the plasma membrane, cilia are compartmentalized organelles whose protein and lipid composition differ from that of the cell body. This compartmentalization is essential for ciliary function and is brought about by microtubule motor-based intraflagellar transport (IFT) and by structural barriers located at the transition zone (TZ) between the basal body and cilium proper, together regulating trafficking of specific proteins in and out of cilia to control their composition^8^,^9^. Consequently, mutations that affect IFT or ciliary TZ integrity are associated with ciliopathies such as Nephronophthisis (NPHP), Bardet Biedl (BBS), Joubert (JBTS) and Meckel Gruber (MKS) syndromes^5^,^8^. The IFT system consists of large ‘trains' of IFT particles with associated ciliary cargoes, which are ferried across the TZ from the base to the tip of cilia by kinesin-2 motors and returned to the base by cytoplasmic dynein 2. Since cilia are devoid of protein synthesis, their assembly and maintenance rely on IFT-mediated transport of axonemal components from the cell body to the ciliary tip where axoneme assembly occurs. Consequently, mutations in IFT components usually lead to absent or structurally defective cilia that are functionally impaired, depending on the protein mutated and the severity of the mutation^9^. IFT has also been implicated directly in ciliary membrane protein trafficking and signalling. For example, during Shh signalling, which in vertebrates functions exclusively at the primary cilium^2^, IFT and a complex of associated BBS proteins (BBSome (ref. 10)) are required for ciliary exit of the Shh receptor Patched homolog 1 (PTCH1), which leaves the ciliary compartment upon binding of Shh, facilitating ciliary entry of Smoothened (SMO) and leading to pathway activation^11^,^12^,^13^. On the other hand, ciliary entry of SMO and additional membrane proteins may occur independently of IFT, for example by lateral diffusion from the plasma- and periciliary membranes across the TZ (refs 14, 15, 16, 17, 18). Despite intense investigation (reviewed in refs 6, 8), the precise mechanisms involved in targeting and transport of most ciliary membrane receptors, from their site of synthesis in the cell body, across the TZ and into the cilium proper, remain unclear. Interestingly, studies in nematodes have implicated kinesins other than conventional anterograde IFT kinesin-2 motors in ciliary membrane protein transport. Specifically, in the male sensory cilia of *Caenorhabditis elegans*, kinesin-3 motor KLP-6 mediates anterograde transport of polycystin-2 (PC-2) towards the ciliary tip, and in a *klp-6* mutant PC-2 signalling is deregulated resulting in male mating behavioural defects^19^.

The kinesin-3 family is one of the largest within the kinesin superfamily of microtubule motors. The mouse genome harbours eight kinesin-3 genes (*Kif1A, Kif1B, Kif1C, Kif13A, Kif13B, Kif14, Kif16A, Kif16B*) while humans contain only seven due to the presence of a single *KIF16* gene^20^. Kinesin-3 motors have been implicated in multiple physiological processes, including transport of organelles and vesicles towards the plus end of microtubules^20^, but so far cilia-related functions have not been described for any mammalian kinesin-3 motor.

In this study we show that kinesin-3 motor KIF13B localizes to centrosomes and primary cilia in mammalian cells and we identify KIF13B as a novel member of the RPGRIP1N-C2 domain-containing TZ protein family that interacts with the ciliary TZ protein Nephrocystin-4 (NPHP4). Using genetic silencing and gene knock out in cultured mammalian cells, we provide evidence that KIF13B and NPHP4 are both required for establishment of a specialized caveolin-1 (CAV1) membrane microdomain at the ciliary TZ, which is essential for Shh-induced accumulation of SMO in the primary cilium as well as for activation of GLI-mediated target gene expression. Our study thus identifies KIF13B as a novel regulator of TZ configuration, ciliary membrane composition and Shh signalling.

## Results

### KIF13B localizes to the basal body and primary cilium

**Figure 1:**
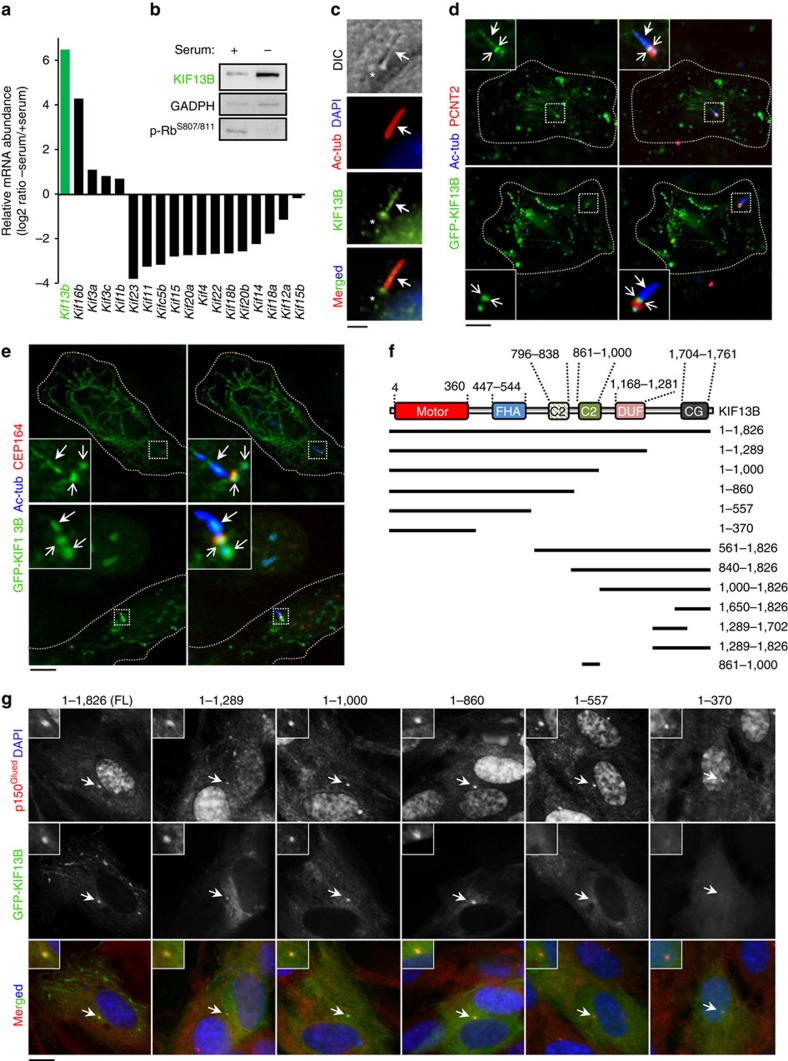
*Kif13b* is upregulated during serum deprivation and localizes to the base and along the primary cilium. (**a**) Kinesins identified in a global transcriptomics analysis of up- and downregulated genes in serum-deprived relative to non-starved NIH3T3 cells. Only kinesin genes that are significantly up- or downregulated (*P*<0.05, two-tailed *t*-test; *n*=3) are listed. (**b**) Immunoblot analysis of MEFs cultured in the presence or absence of serum using mouse monoclonal KIF13B antibody #1 (see Methods for details). (**c**) Localization of endogenous KIF13B in serum-starved MEFs using KIF13B-specific rabbit antiserum (green) (Supplementary Fig. 1a) and antibody against acetylated α-tubulin (Ac-tub; red) to mark the cilium (closed arrow). DNA is stained with DAPI (blue). Note localization of KIF13B along the length and base (asterisk) of the cilium. (**d**) GFP-KIF13B (green) localization in pre-extracted RPE1 cells. GFP-KIF13B is found along the cilium (Ac-tub, blue) and at two distinct puncta at the ciliary base, flanking Pericentrin 2 (PCTN2; red). Open arrows indicate the distal end of the basal body; closed arrows show cilia. Quantitative analysis revealed that approximately 72% of the cells displayed GFP-KIF13B at the ciliary base whereas in 23% of the cells GFP-KIF13B was detected both at the ciliary base and along the axoneme (33 cells analysed in total, *n*=3). (**e**) GFP-KIF13B co-localizes with CEP164 in pre-extracted RPE1 cells. Cilia and CEP164 were stained with the indicated antibodies. Open arrows indicate the two different pools of GFP-KIF13B at the basal body; closed arrows show cilia. (**f**,**g**) Schematics of the GFP-tagged deletion constructs and IFM analysis (representative images) of their ability to localize to the centrosome, marked by p150^Glued^ antibody (red), in non-ciliated interphase RPE1 cells. Assessment of centrosome localization, quantified in Supplementary Fig. 1h, is based on IFM analysis of at least 40 cells per construct (*n*=3). DNA was stained with DAPI (blue). Insets in (**g**) show enlarged views of the centrosome region. Scale bars: 2 μm in **c**; 10 μm in **d**,**e**,**g**.

To identify putative kinesins regulating ciliary trafficking in mammals we performed global transcriptomics profiling of mouse NIH3T3 cells cultured in the presence or absence of serum to induce ciliogenesis, reasoning that genes with ciliary functions are likely upregulated during ciliogenesis. Among the kinesin gene transcripts that were highly upregulated by serum deprivation, we identified *Kif13b* (also known as GAKIN) as the most abundant (Fig. 1a), confirming an earlier qPCR-based screen^21^. Furthermore, luciferase assays in NIH3T3 cells using wild type and mutant *Kif13b* promoter constructs substantiated that *Kif13b* is transcriptionally upregulated by serum deprivation, and that this upregulation involves at least three different sites in its promoter (Supplementary Fig. 1b–d). Finally, immunoblot analysis of mouse embryonic fibroblasts (MEFs) demonstrated that KIF13B protein is correspondingly upregulated during serum deprivation (Fig. 1b). Consequently we chose *Kif13b* for further analysis.

*Kif13b* encodes a vertebrate kinesin-3 motor protein that, together with its proximal paralog KIF13A (comprising the KIF13 subgroup) (Supplementary Fig. 2a), shows the closest sequence homology to *C. elegans* KLP-6 (see below) previously implicated in ciliary trafficking and sensory functions^19^. To investigate whether KIF13B similarly regulates trafficking at the primary cilium, we first asked whether it localizes to this compartment. Indeed, immunofluorescence microscopy (IFM) and live cell imaging analysis revealed that endogenous and GFP-tagged KIF13B accumulated prominently at the ciliary base and in some instances along the cilium (Fig. 1c-e; Supplementary Fig. 1e). These observations are in line with previous reports identifying KIF13B in the proteome of swine choroid plexus^22^ or MEF (ref. 23) 9+0 cilia. Close examination of GFP-KIF13B localization in ciliated detergent-extracted, fixed Human Telomerase-Immortalized retinal pigmented epithelial (hereafter: RPE1) cells indicated that the pool of GFP-KIF13B at the ciliary base is concentrated at two distinct sites flanking the centrosomal marker Pericentrin 2 (PCTN2), with the most distal site overlapping the mother centriole distal appendage/transition fibre marker CEP164 (Fig. 1d,e). Quantitative analysis of detergent-extracted, GFP-KIF13B expressing cells (33 cells analysed in total) revealed that approximately 72% of the cells displayed GFP-KIF13B at the ciliary base whereas in 23% of the cells GFP-KIF13B was detected both at the ciliary base and along the axoneme (*n*=3). GFP-KIF13B was also found to localize to the centrosome in non-ciliated cells (Fig. 1g; Supplementary Fig. 1h), as well as to the cytoplasmic microtubule network (Supplementary Fig. 1f), consistent with previous reports^24^,^25^,^26^. Thus, KIF13B is upregulated by serum deprivation and localizes in part to the primary cilium-centrosome axis.

### KIF13B tail region contains two RPGRIP1N-C2 type domains

**Figure 2:**
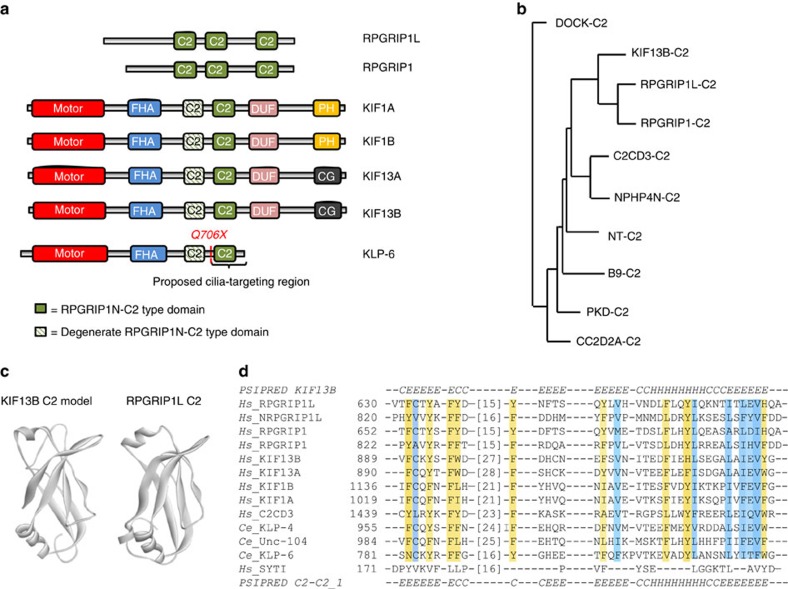
Identification of RPGRIP1N-C2 type domains in KIF13B and related kinesin-3 motors. (**a**) Schematics of the domain structure of selected RPGRIP1N-C2 domain-containing proteins identified by bioinformatics analysis. (**b**) Cladogram showing the evolutionary relationship of the different C2 domain families and superfamilies. DOCK-C2, CC2D2A-C2, B9-C2, NT-C2 represent PFAM C2 superfamilies. NPHP4N-C2, human NPHP4 central C2 domain (amino acids 661-756); C2CD3, human C2CD3 C-terminal C2 domain (amino acids 1,436-1,501); RPGRIP1, human RPGRIP1 RPGRIPN-C2 type domain (amino acids 609-711); RPGRIP1L, human RPGRIP1L RPGRIP1N-C2 type domain (amino acids 588-690); KIF13B-C2, human KIF13B RPGRIP1N-C2 type domain (amino acids 861-1,000). The cladogram was reconstructed using a Neighbour Joining Using PAM250 method implemented in the Jalview program under default parameters. (**c**) Predicted three-dimensional structure of KIF13B-C2^861-1,000^ compared to the solved crystal structure of the RPGRIP1N-C2 domain in RPGRIP1L (PDB: 2YRB). (**d**) Alignment of selected RPGRIP1N-C2 type domains identified in our bioinformatic survey. The remote C2 domain of synaptotagmin I (SYTI) is included for comparison. The defining signature of aromatic amino acids specific to the RPGRIP1N-C2 type domains are highlighted in light orange. Homologous residues other than those found in aromatic amino acid profiles are shown in blue. Secondary structure predictions as assessed by PSIPRED are shown above (predicted for KIF13B RPGRIP1N-C2 domain) and below (predicted for RPGRIP1 RPGRIP1N-C2 domain) the alignment. H, α-helix; E, β-sheet; C, random coils.

To delineate the minimal polypeptide portion required for centrosomal localization of KIF13B, cells expressing different GFP-KIF13B truncations (Fig. 1f; Supplementary Fig. 1g) were analysed by IFM and the data quantified. This analysis showed that centrosomal accumulation of GFP-KIF13B was mediated by one or more sites located in the tail region spanning from residues 558-1,649 (Fig. 1f,g; Supplementary Fig. 1h). Interestingly, computational sequence analysis revealed that this region (residues 861–1,000) contains an RPGRIP1N-C2 type domain (also known as C2-C2_1 domain; registered as DUF3250 in the PFAM database) (E-value=2.6e–06), previously identified exclusively in the TZ components RPGRIP1 (also known as LCA6) and RPGRIP1L (also known as NPHP8/FTM/MKS5) (ref. 27) (Fig. 2a,d; Supplementary Fig. 2b), thus expanding the evolutionary sub-branch of RPGRIP1N-C2 domains previously classified by Zhang and Aravind (Fig. 2b) (ref. 27). Homology searches in the Protein Data Bank ( www.rcsb.org 1999) identified the solved RPGRIP1N-C2 domain as the best match to this KIF13B C2 domain (hereafter: KIF13B-C2^861-1,000^), allowing 3D structure modelling of KIF13B-C2^861-1,000^ (Fig. 2c). Comparative analysis of secondary structure scores, as assessed by three-state PSI-blast based secondary structure PREDiction (PSIPRED), between KIF13B-C2^861-1,000^ and the RPGRIP1N-C2 profile yielded highly significant HHpred probabilities in the range of (97-98) (ref. 28), and visual inspection of the aligned sequences indicated near perfect agreement between the predicted secondary structures (Fig. 2d), supporting the sequence homology searches. A smaller, more degenerate RPGRIP1N-C2 type domain was also identified N-terminal to this domain in KIF13B (residues 796-838; Fig. 2a). Interestingly, while KIF13B-C2^861-1,000^ showed closest homology by sequence among all C2 domains to the RPGRIP1N-C2 domain in the PFAM database (Fig. 2a,b; Supplementary Fig. 2b), significant homology was also identified to the neighbouring PKC-C2-like domains in the RPGRIP1/L proteins, indicating that both of the central C2 domains, RPGRIP1N-C2 and PKC-C2, have hitherto unknown sequence homology. Indeed, we could show that the RPGRIP1N-C2 and PKC-C2 domains in RPGRIP1/1L and KIF13B-C2^861-1,000^ bear a distinct signature of enriched conserved aromatic residues (Fig. 2d and Supplementary Fig. 2b) not found in the other C2 families, including the PKC-C2 subfamily. In addition, extensive database searches for other proteins bearing this domain signature identified three additional human proteins: the kinesin-3 members KIF13A, KIF1A, KIF1B, as well as *C. elegans* kinesin-3 members KLP-4, KLP-6 and UNC-104 (Fig. 2a; Supplementary Fig. 2a,b), thus establishing a novel/expanded family of RPGRIP1N-C2 domain-containing proteins (Fig. 2b,d). When using the *C. elegans* KLP-6 RPGRIP1N-C2 domain (amino acids 701-824) as a query we also found more remote homology to C2 domains in C2CD3 (E-value=0.01), supporting that C2CD3 is related to the RPGRIP1N-C2 domain branch (Fig. 2b and ref. 27). KLP-6 mediates ciliary membrane protein trafficking in *C. elegans* male sensory neurons, and a mutant expressing C-terminally truncated KLP-6 lacking the identified RPGRIP1N-C2 domain (residues 701-824; Fig. 2a), rendered KLP-6 unable to accumulate in cilia, causing male mating defects^19^. Therefore, this RPGRIP1N-C2 domain likely has a ciliary function in this kinesin.

Given that the kinesin-3 members KIF13A, KIF1A, KIF1B also contain RPGRIP1N-C2 domains, like KIF13B (see above), we tested whether these kinesin-3 motors similarly localize to the cilium-centrosome axis. Expression of fluorescently tagged versions of these motors in ciliated RPE1 cells, followed by IFM analysis of pre-extracted, fixed cells, revealed undetectable levels of heterologously expressed KIF13A at the centrosome/cilium whereas in approximately 20-25% of cells expressing KIF1A or KIF1B, the fusion protein was detected at the ciliary base but not within the cilium itself (Supplementary Fig. 3a,b). Hence KIF13B displays a unique preference for the cilium-centrosome axis.

### KIF13B binds NPHP4 via its C2 domain and DUF region

**Figure 3:**
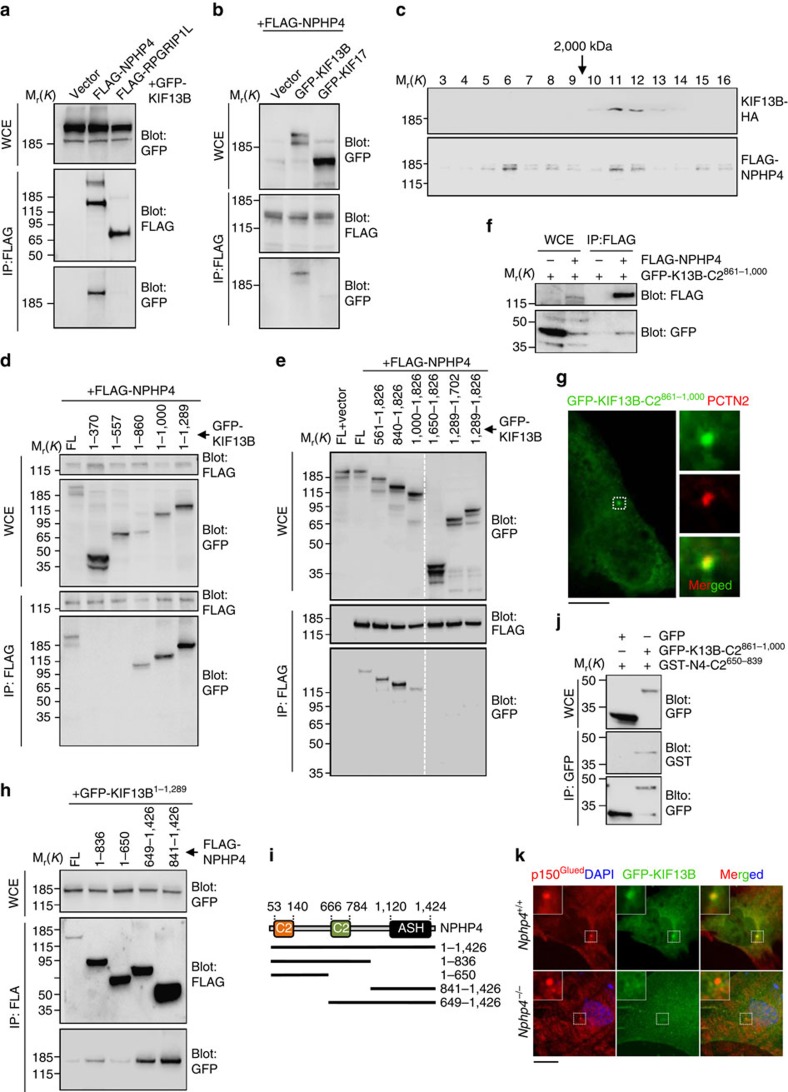
KIF13B interacts with NPHP4 via its C2 domain and DUF region. (**a**) FLAG pulldown analysis of HEK293T cells co-expressing GFP-KIF13B with FLAG-NPHP4 or FLAG-RPGRIP1L. FLAG-constructs were IP'ed using anti-FLAG conjugated agarose beads and resolved proteins were subjected to immunoblot analysis with the indicated antibodies. WCE, whole cell extract. The appearance of GFP-KIF13B as a double band (top panel) is likely a consequence of protein overexpression. (**b**) FLAG IP of cell extracts from HEK293T cells co-expressing FLAG-NPHP4 with either GFP-KIF13B or GFP-KIF17 using anti-FLAG conjugated beads followed by immunoblotting with indicated antibodies. (**c**) Size exclusion chromatography of HEK293T cells co-expressing KIF13B-HA and FLAG-NPHP4 followed by immunoblot analysis with HA or FLAG antibodies. (**d**,**e**) FLAG-NPHP4 expressing HEK293T cells co-expressing GFP-tagged full-length (FL) or truncated KIF13B fusion proteins were subjected to FLAG IP followed by immunoblotting with indicated antibodies. (**f**) IP of HEK293T cells co-expressing GFP-KIF13B-C2^861-1,000^ and FLAG-NPHP4 using antibodies as indicated. (**g**) IFM of RPE1 cells expressing GFP-KIF13B-C2^861-1,000^. The insets show enlarged views of the boxed area containing the centrosome, marked by PCTN2 antibody. (**h**) HEK293T cells co-expressing GFP-KIF13B and truncated versions of FLAG-NPHP4, as indicated, were harvested and cell extracts subjected to FLAG IP followed by immunoblotting with indicated antibodies. (**i**) Diagram showing FLAG-NPHP4 deletion constructs used in **h**. (**j**) Binding assay of immuno-purified GFP (negative control) or GFP-KIF13B-C2^861-1,000^ mixed with bacterially purified GST fused to the central C2 domain of NPHP4 (GST-N4-C2^2650-839^). (**k**) IFM analysis of *Nphp4*^+/+^ and *Nphp4*^−/−^ MEFs expressing GFP-KIF13B (green). The insets show enlarged views of the boxed area containing the centrosome, marked by antibody against p150^Glued^ (red). In all, 45-49 cells were analysed per condition (*n*=2). Difference in the percentage of cells with GFP-KIF13B centrosome localization obtained as mean±s.e.m. (*n*=2) *Nphp4*^+/+^; *Nphp4*^−/−^ (88.5±3.5; 42±3) and two-tailed *t*-test (*P*<0.01). Representative IFM pictures of MEFs are shown. Scale bars: 10 μm in **g**,**k**.

RPGRIP1 and RPGRIP1L reside at the centrosome and TZ where they have been shown to anchor the second C2 domain of NPHP4 via mutual C2-C2 domain dimerizations^29^,^30^. We therefore asked whether KIF13B is recruited to or tethered at centrosomes through physical binding to any of these C2 domain-containing TZ proteins. Indeed, an immunoprecipitation (IP) assay revealed that KIF13B interacts with NPHP4 but not with RPGRIP1L (Fig. 3a). The interaction between KIF13B and NPHP4 appeared to be specific since the ciliary kinesin-2 motor KIF17 did not interact with NPHP4 under similar conditions (Fig. 3b). However, we also detected interaction between NPHP4 and KIF1B (Supplementary Fig. 3c), suggesting that NPHP4 can bind to other C2 domain-containing kinesin-3 motors. Reciprocal IP of GFP-KIF13B confirmed its binding to NPHP4 (Supplementary Fig. 3d) and size exclusion chromatography of extracts from KIF13B-HA and FLAG-NPHP4 expressing cells indicated that NPHP4 forms several larger complexes, one of which co-elutes with KIF13B in a high molecular mass fraction (Fig. 3c). This substantiates that KIF13B and NPHP4 reside in a mutual complex and that NPHP4 exists in multiple complexes as proposed recently by others^31^. Furthermore, IP analyses of GFP-KIF13B deletion fragments revealed that the KIF13B C2 domain-containing region mediates interaction with NPHP4 (Fig. 3d,e), but that an additional NPHP4 binding site is present within the region spanning residues 1,000–1,288 of KIF13B (Fig. 3e), which contains a domain of unknown function (DUF3694 (DUF); Fig. 1f). IP and IFM analysis with tagged KIF13B-C2^861-1,000^ confirmed its sufficiency for NPHP4 binding (Fig. 3f) and centrosome localization (Fig. 3g). The observed interaction of NPHP4 with KIF13B (Fig. 3d,e) was not a result of spurious protein-protein interactions caused by protein truncations, as the same truncations failed to bind another FLAG-tagged protein unrelated to NPHP4 (Angiomotin p80; Supplementary Fig. 3f). Reciprocal mapping of the KIF13B binding domain in NPHP4 showed that the central C2 domain (NPHP4-C2^666-784^) (ref. 27), but not the divergent N-terminal C2 domain (residues 53-140; ref. 27), mediates KIF13B binding. An additional KIF13B-binding domain was identified in the NPHP4 C-terminus harbouring ASPM, SPD-2, Hydin (ASH) domains (Fig. 3h,i), whereas a binding assay using bacterially purified GST-NPHP4-C2^650-839^ fusion protein and high salt immunoisolated GFP-KIF13B-C2^861-1,000^ suggested that KIF13B-C2^861-1,000^ directly binds NPHP4-C2^650-839^ (Fig. 3j). Supporting this, overexpressed FLAG-NPHP4 co-localized with full length GFP-KIF13B in cytoplasmic puncta, whereas no co-localization was observed with a GFP-KIF13B version containing only the motor and FHA domain (Supplementary Fig. 3e). Moreover, expression of GFP-KIF13B in *Nphp4*^+/+^ and *Nphp4*^−/−^ MEFs indicated that NPHP4 is at least partially required for recruitment of GFP-KIF13B to the centrosome (Fig. 3k). Specifically, we observed 88.5% (±3.5%) GFP-KIF13B positive centrosomes in *Nphp4*^+/+^ MEFs versus 42% (±3%) GFP-KIF13B positive centrosomes in *Nphp4*^−/−^ MEFs (*n*=2; 45-49 cells analysed per condition). This suggests that NPHP4 alone or in concert with other scaffolds recruits or tethers KIF13B at the ciliary base. In summary, KIF13B interacts with NPHP4 via its C2 domain-containing and DUF3694 regions and this interaction is at least in part required for localization of KIF13B to the centrosome.

### KIF13B and NPHP4 regulate CAV1 TZ localization

**Figure 4:**
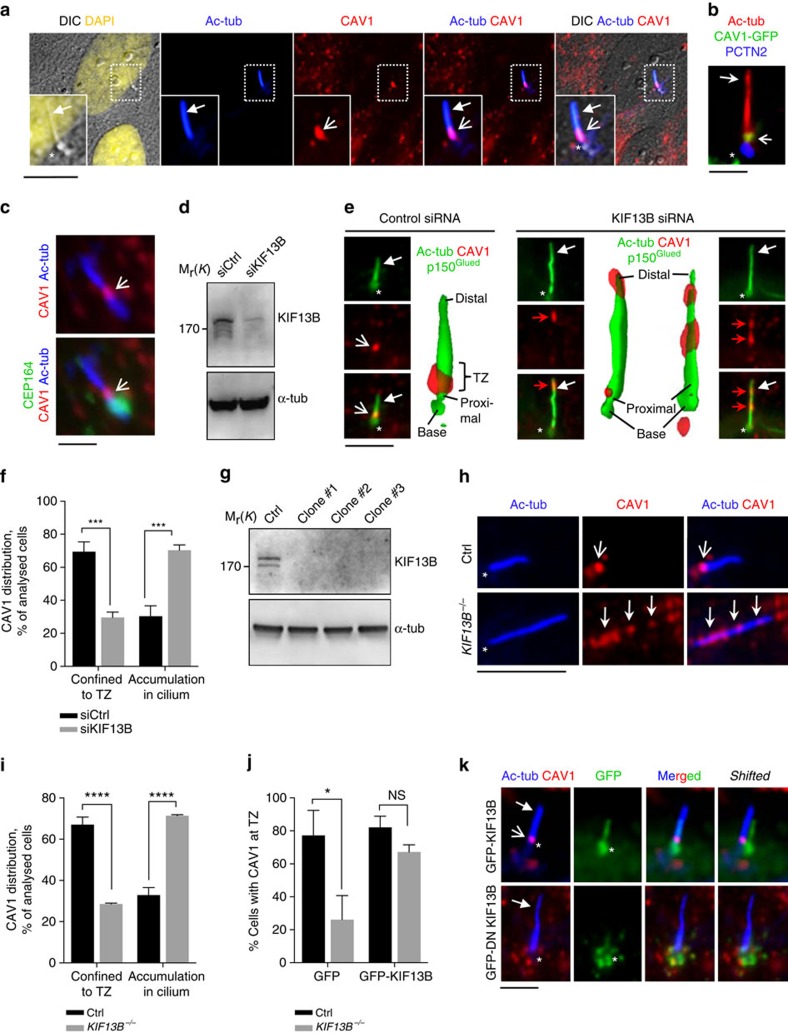
KIF13B-dependent TZ localization of CAV1 in RPE1 cells. (**a**) IFM using antibodies against CAV1 (red) and Ac-tub (blue) to mark the cilium (closed arrows). Open arrow marks CAV1 above the basal body (asterisk). The leftmost image is a digital interference contrast (DIC) micrograph of the cell with the nucleus stained with DAPI (pseudocoloured yellow). Insets show enlargement of the cilium-centrosome region. (**b**) IFM of CAV1-GFP expressing cells. Open arrow, TZ; asterisk, basal body (PCTN2; blue). (**c**) IFM of endogenous CAV1 (red). CEP164 (green) marks the transition fibres. (**d**) Cells treated with control- or KIF13B siRNA were immunoblotted and proteins detected with indicated antibodies. For KIF13B, mouse monoclonal anti-KIF13B #2 was used (see Methods). (**e**) CAV1 staining (red; open arrow) in ciliated cells treated with control- or KIF13B-specific siRNA. Closed arrow marks the cilium (Ac-tub, green), asterisk the centrosome (p150^Glued^ antibody, green). (**f**) Quantification of CAV1 staining in control- or KIF13B-siRNA transfected cells. In all, 50-100 cells were analysed per condition (*n*=3). Bars: mean±s.e.m. *P* values result from two-way ANOVA followed by Sidak's multiple comparison (***, *P*≤0.001). (**g**) Immunoblot of control (wild type) and three KIF13B knock out clones generated using CRISPR-Cas9 technology. Clone #3 was used for further analysis and designated *KIF13B*^−/−^. For KIF13B detection, mouse anti-KIF13B #2 antibody was used (see Methods). (**h**) IFM of CAV1 in control (wild type) and *KIF13B*^−/−^ cells. Open arrow, TZ; closed arrows, cilia. (**i**) Quantification of data in **h**. Between 5 and 20 cells were analysed per condition (*n*=3). Bars: mean±s.e.m. *P* values result from two-way ANOVA followed by Sidak's multiple comparison (****, *P*≤0.0001). (**j**) Quantification of CAV1 staining in control (wild type) and *KIF13B*^−/−^ cells expressing GFP or GFP-KIF13B. Bars: mean±s.e.m. *P* values result from two-way ANOVA followed by Sidak's multiple comparison (*, *P*≤0.05; NS, not significant). Between 5 and 20 cells were analysed per condition (*n*=3). (**k**) CAV1 localization (red; open arrow) in cells expressing GFP-KIF13B or GFP-DN KIF13B (green). Closed arrow: cilium (Ac-tub; blue). Scale bars: 10 μm in **a**; 2 μm in **b**,**c**; 5 μm in **e**,**h**; 3 μm in **k**.

Previous work uncovered a role of KIF13B in conveyance of CAV1, a structural component of caveolae, specialized cholesterol-enriched condensed membrane microdomains^32^, from the plasma membrane to cytoplasmic vesicles^33^. Because *Chlamydomonas reinhardtii* NPHP4 resides in the distal TZ in close proximity to the TZ membrane^34^, which comprises a highly condensed membrane microdomain^35^ similar to caveolae^36^, we surmised that KIF13B may regulate the localization of CAV1 to the ciliary membrane compartment or in the TZ. Remarkably, both endogenous and GFP-tagged CAV1 were detected at the ciliary compartment of RPE1 cells and found to be enriched at the TZ region, distal to CEP164, which marks the distal mother centriole appendages/basal body transition fibres (Fig. 4a–c; Supplementary Fig. 4a). A similar localization was observed for another marker of condensed membrane microdomains, flotillin-2 (FLOT2; Supplementary Fig. 4b), providing further support for the condensed membrane microdomain in the TZ. Treatment with the cholesterol depleting reagent methyl-β-cyclodextrin (MBCD) led to removal of CAV1 from the TZ area without affecting ciliary localization of type III adenylyl cyclase (ACIII) and inositol polyphosphate 5-phosphatase E (INPP5E) (Supplementary Fig. 4c-e), suggesting that the CAV1-enriched microdomain in the TZ is enriched in sterols. Furthermore, because ciliary localization of INPP5E was reported to be disrupted by mutations in TZ proteins such as TMEM231 (also known as JBTS20 and MKS11) (ref. 37) and MKS1 (also known as BBS13) (ref. 38), the results suggest that removal of CAV1 does not grossly perturb TZ structure and function. Similar to MBCD treatment, transfection of RPE1 cells with CAV1-specific siRNA also led to removal of CAV1 antibody staining at the TZ (Supplementary Fig. 4f), validating the specificity of the CAV1 antibody.

**Figure 5:**
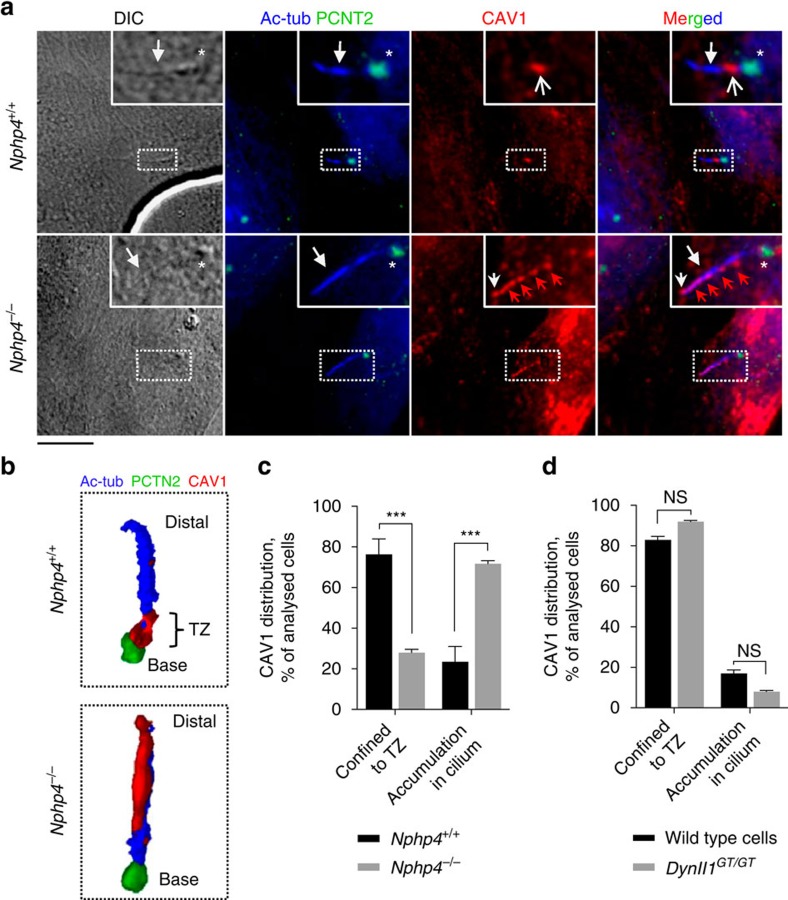
CAV1 TZ localization is disrupted in cells lacking NPHP4. (**a**,**b**) IFM analysis of wild type (*Nphp4*^+/+^) and *Nphp4*^−/−^ MEFs stained with CAV1 (red; open arrow) and PCTN2 (green; asterisk) antibodies. (**c**,**d**) Quantification of CAV1 staining patterns in *Nphp4*^+/+^ and *Nphp4*^−/−^ MEFs (**c**), and in wild type and *Dynll1*^GT/GT^ MEFs (**d**). At least 30 cells were analysed per condition (*n*=3). Bars represent mean±s.e.m. *P* values result from two-way ANOVA followed by Sidak's multiple comparison (***, *P*≤0.001; NS, *P*≥0.05, not significant). Scale bar: 5 μm.

Interestingly, in cells depleted for KIF13B by siRNA treatment (Fig. 4d) CAV1 was largely absent from the TZ and accumulated aberrantly along and near the distal region of the cilium (Fig. 4e,f). To support these results, we generated three KIF13B knock out RPE1 cell lines using CRISPR-Cas9 technology^39^ (Fig. 4g) and chose one of these (clone #3; hereafter referred to as *KIF13B*^−/−^) for further analysis. Importantly, IFM analysis of CAV1 localization in *KIF13B*^−/−^ and control RPE1 cells confirmed that CAV1 TZ confinement is reduced in *KIF13B*^−/−^cells and *KIF13B*^−/−^cells expressing GFP (Fig. 4h-j), whereas TZ confinement of CAV1 is restored in *KIF13B*^−/−^cells complemented with wild-type GFP-KIF13B (Fig. 4j). These results suggest that CAV1 mislocalization in KIF13B-ablated cells can be rescued by heterologous KIF13B expression. Analysis of cells expressing dominant negative GFP-KIF13B (hereafter: GFP-DN KIF13B) in which the motor domain has been deleted^26^ led to sequestration of CAV1 in GFP-DN KIF13B positive puncta in the cytoplasm and in the vicinity of the basal body, correlating with CAV1 absence from the TZ region (Fig. 4k; Supplementary Fig. 4g). These puncta likely correspond to endosomes as revealed by co-expression and staining with the early endosome marker Myc-RAB5 or a constitutively active Myc-RAB5 mutant (Myc-CA RAB5) (Supplementary Fig. 5a). Notably, GFP-KIF13B did not seem to co-localize with CAV1 at the TZ (Fig. 4k), consistent with the observed co-localization of GFP-KIF13B with CEP164 (Fig. 1e; see also Fig. 4c). Collectively, based on three different approaches to inhibit KIF13B activity (siRNA depletion, CRISPR-Cas9-mediated gene knock-out and rescue experiments, and expression of dominant negative fusion protein), we conclude that KIF13B is required for establishment of a CAV1 microdomain at the TZ. Finally, consistent with a role of NPHP4 in recruiting or tethering KIF13B at the centrosome (Fig. 3k and see text above), the ciliary CAV1 mislocalization seen in KIF13B invalidated cells (Fig. 4e-k) was essentially phenocopied in *Nphp4*^−/−^ MEFs, but not in *Nphp4*^+/+^ MEFs (Fig. 5a-c). In contrast *Dynll1*^GT/GT^ MEFs, which have a mild defect in retrograde IFT (ref. 40), displayed normal ciliary/TZ CAV1 localization (Fig. 5d).

We did not observe co-localization of GFP-DN KIF13B with EEA1 (Supplementary Fig. 5b), which marks a subset of early endosomes, or with PCM1, which marks centriolar satellites (Supplementary Fig. 5c). Expression of GFP-DN KIF13B did not markedly impair localization of the ciliary membrane component ARL13B (Supplementary Fig. 5d) nor the IFT-B protein IFT88 (Supplementary Fig. 5e), indicating that KIF13B specifically affects ciliary localization of CAV1. Further, because ciliary localization of ARL13B was reported to be disrupted by mutations in several TZ proteins, including TMEM231 (ref. 37), MKS1 (ref. 38), NPHP4 and others^41^, the results indicate that expression of GFP-DN KIF13B does not grossly perturb ciliary TZ structure and function.

### KIF13B and CAV1 promote Shh-induced ciliary SMO accumulation

**Figure 6:**
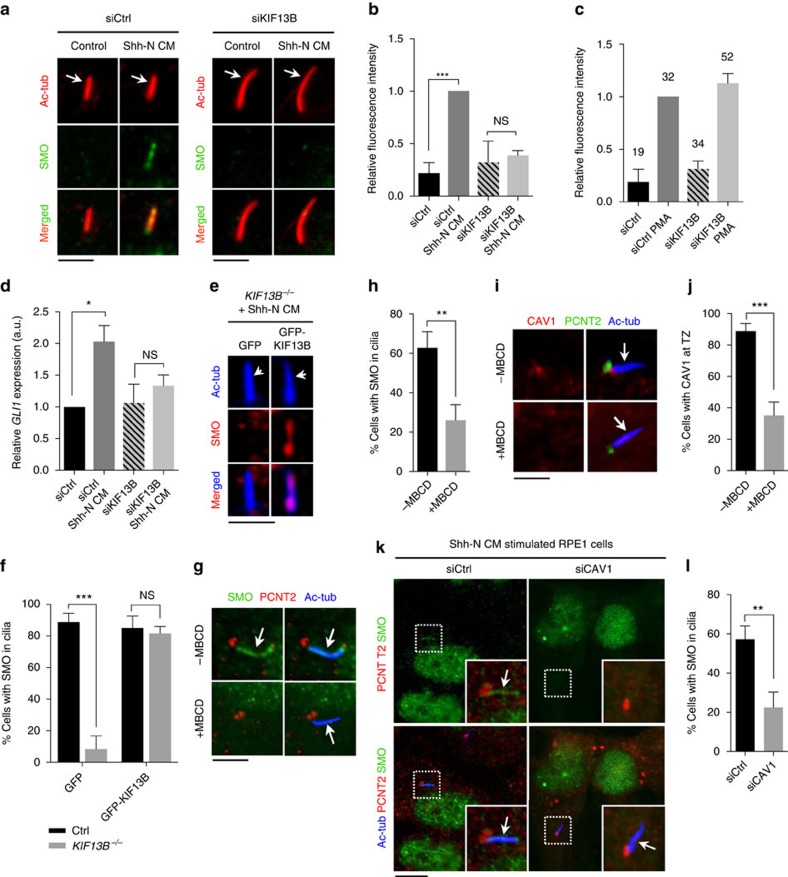
Defective SMO ciliary translocation and Shh signalling in KIF13B- or CAV1-deficient cells. (**a**) IFM showing ciliary accumulation of SMO (green) in control-siRNA transfected cells stimulated with Shh-N CM for 24 h (left panels). In KIF13B-depleted cells, Shh-N CM did not induce detectable ciliary accummulation of SMO (right panels). The cilium was stained with Ac-Tub antibody (red, arrows). (**b**) Quantification of the data in **a**. The relative fluorescence intensity of SMO ciliary staining was measured (see Methods for details) for at least 50 cells per condition (*n*=3). Bars represent mean±s.e.m. *P* values result from a two-tailed *t*-test (***, *P*≤0.001; NS, *P*≥0.05, not significant). (**c**) Relative fluorescence intensity of SMO ciliary staining in control or KIF13B-depleted cells stimulated with purmorphamine (PMA). Total number of cilia measured per condition is indicated above each column. Bars represent mean±s.e.m. (*n*=2). (**d**) Depletion of KIF13B leads to reduced *GLI1* expression in Shh-N CM stimulated cells, as determined by RT-qPCR analysis. The analysis was performed on serum starved RPE1 cells treated with Shh-N CM for 24 h using *B2M* as a reference gene (*n*=3). Bars represent mean±s.e.m. *P* values result from a two-tailed *t*-test (*, *P*≤0.05). (**e**) IFM analysis of ciliary SMO staining (red) in Shh-N CM stimulated *KIF13B*^−/−^ RPE1 cells expressing GFP alone or GFP-KIF13B, as indicated. The cilium is stained with Ac-tub antibody (blue, arrows). (**f**) Quantification of data shown in **e**. Bars represent mean±s.e.m. *P* values result from two-way ANOVA followed by Sidak's multiple comparison (***, *P*≤0.001; NS, *P*≥0.05, not significant). Between 5 and 20 cells were analysed per condition (*n*=3). (**g**,**i**,**k**) IFM analysis of Shh-N CM induced ciliary SMO (green) localization in RPE1 cells with and without treatment with MBCD (**g**,**i**) or CAV1-specific siRNA (**k**). The cilium and centrosome are stained with antibodies against Ac-tub (blue, arrows) and PCTN2 (red), respectively. (**h**,**j**,**l**) Quantification of data in (**g**,**i**,**k**), respectively. Between 30 and 40 cells were analysed per condition (*n*=3). Bars represent mean±s.e.m. *P* values result from two-tailed *t*-test (***, *P*≤0.001; **, *P*≤0.01). Scale bars: 5 μm.

We next addressed the functional significance of KIF13B at cilia by investigating effects of KIF13B invalidation on Shh signalling, a cilium-dependent pathway^42^ that is initiated by Shh-induced clearance of the Shh receptor PTCH1, which in turn leads to ciliary accumulation of SMO and activation of GLI-dependent transcription^2^,^11^,^12^. We chose to focus on this pathway because SMO ciliary translocation is partially dependent on NPHP4 (ref. 43) and is impaired by MBCD-induced cholesterol depletion^44^. Shh signalling is known to be important in the maintenance of retinal pigmented epithelium from which the RPE1 cell line is derived^45^, and we therefore applied our analysis in RPE1 cells, in which we already established a requirement for KIF13B in mediating CAV1 TZ localization (Fig. 4e-k). In control RPE1 cells, stimulation with conditioned medium (CM) from cell cultures expressing N-terminal Shh (Shh-N) led to pronounced ciliary enrichment of SMO (Fig. 6a,b; Supplementary Fig. 3g) and increased *GLI1* expression (Fig. 6d), as expected. In contrast, we found a severe reduction in Shh-N CM-induced ciliary SMO accumulation and *GLI1* transcript levels in KIF13B siRNA-depleted cells (Fig. 6a,b,d), whereas purmorphamine-mediated ciliary accumulation of SMO was unaffected by KIF13B knock down (Fig. 6c). Given that purmorphamine directly targets and activates SMO independently of PTCH1 (ref. 46), we infer from this result that KIF13B-mediated effects on SMO ciliary translocation are PTCH1-dependent. To confirm that KIF13B promotes Shh-mediated ciliary translocation of SMO, we performed IFM analysis of SMO ciliary localization in Shh-N CM stimulated control RPE1 and *KIF13B*^−/−^ cells expressing either GFP-KIF13B or GFP alone. This analysis showed that upon Shh-N CM stimulation, GFP-KIF13B expression, but not GFP alone, could rescue SMO accumulation in cilia compared to control conditions (Fig. 6e,f), substantiating a requirement for KIF13B in Shh-induced SMO ciliary accumulation. Since treatment with MBCD (Fig. 6g-j; ref. 44) or CAV1 depletion (Fig. 6k,l; Supplementary Fig. 4f) similarly prevented Shh-N CM-induced ciliary SMO accumulation, these results suggest that KIF13B promotes ciliary SMO translocation indirectly via establishment of a CAV1 membrane microdomain at the TZ compartment.

### KIF13B promotes WNT5A expression and ciliary elongation

Since CAV1-mediated effects on membrane lipid composition impinge on a range of additional signalling pathways, such as receptor tyrosine kinase (for example, IGF-1R, PDGFRα, EGFR) and WNT signalling^32^, which may also be coordinated by primary cilia^3^,^4^ we reasoned that KIF13B-mediated control of ciliary CAV1 homeostasis might affect ciliary function beyond Shh signalling. Indeed, during the course of our studies, we noticed that KIF13B-deficient cells (for example, Fig. 4e) had elongated cilia. Ciliary length measurements of such cells (Supplementary Fig. 6a) or cells expressing GFP-DN KIF13B (Supplementary Fig. 6b) confirmed this observation. Ciliogenesis frequency appeared to be the same in control and KIF13B siRNA-depleted cells (Supplementary Fig. 6c). Similarly, in CAV1 siRNA-depleted cells ciliation frequency was not significantly different from that of control cells (Supplementary Fig. 6d), but the cilia appeared to be longer (Supplementary Fig. 6e).

The mechanisms that control ciliary length are highly complex^47^, and KIF13B could therefore regulate ciliary length by multiple direct and/or indirect mechanisms. Because previous work suggested a role for low density lipoprotein receptor-related protein 1 (LRP1) in regulating expression of the WNT ligand WNT5A (ref. 48), a negative regulator of ciliary length^49^, and since KIF13B was reported to interact with and promote endocytosis of LRP1 (ref. 33) we tested if the elongated ciliary length phenotype observed in KIF13B-depleted cells was due to reduced expression of WNT5A. Indeed, RT-qPCR analysis of KIF13B depleted cells showed a significant reduction in WNT5A transcript levels as compared to control siRNA-treated cells (Supplementary Fig. 6f). Moreover, stimulation with purified WNT5A ligand completely rescued the ciliary length phenotype of KIF13B-depleted cells (Supplementary Fig. 6g). Therefore we conclude that KIF13B negatively regulates ciliary length, at least in part, by promoting expression of WNT5A, although alternative pathways cannot be excluded.

## Discussion

The TZ plays an essential role in regulating ciliary protein and lipid composition, which is critical for establishment and maintenance of the cilium as a compartmentalized signalling organelle. Consequently, genes that encode components of the TZ are frequently mutated in ciliopathies, such as NPHP, JBTS and MKS (refs 5, 8). These TZ proteins interact extensively with each other, forming two distinct subcompartments/modules within the TZ region known respectively as the NPHP and MKS/JBTS modules^8^,^31^,^50^. RPGRIP1, RPGRIP1L and NPHP4 are integral constituents of the NPHP TZ module and were shown previously to harbour distinct RPGRIP1N-C2 type domains^27^ that are important for their mutual interaction and mutated in ciliopathies such as JBTS, NPHP and Leber congenital amaurosis^29^,^30^. Here we have identified KIF13B as a new member of the RPGRIP1N-C2 domain-containing protein family and we have uncovered a physical interaction between KIF13B and NPHP4, which is mediated in part by the KIF13B C2 domain-containing region as well as by the DUF3694 region located between residues 1,168 and 1,281. This suggests that the KIF13B-NPHP4 interaction is complex and further experiments will be required to elucidate the precise binding mechanism involved. However, given that KIF13B is known to exist in an autoinhibitory conformation in which the tail region folds back on the motor domain^51^, it can be speculated that NPHP4 binds the autoinhibited version of KIF13B and contributes to regulation of its activity. This would explain why NPHP4 associates with multiple regions in the KIF13B tail.

Since we did not observe physical interaction between KIF13B and RPGRIP1L, KIF13B appears to reside in a distinct NPHP4-containing complex that excludes RPGRIP1L. Our IFM and live cell imaging analyses indicated that the pool of KIF13B associated with the ciliary base is concentrated primarily at the proximal region of the TZ marked by CEP164. Interestingly, a recent study in *C. elegans* showed that NPHP4 not only localizes to the distal end of the TZ, but also concentrates at the proximal end coinciding with CEP164 (ref 31, 34). Using *Nphp4*^−/−^ mutant MEFs we found that NPHP4 is at least partially required for recruitment or tethering of KIF13B to the centrosome/basal body, consistent with our results showing that the KIF13B truncations that bind NPHP4 (Fig. 3d,e) also localize prominently to the centrosome (Fig. 1g and Supplementary Fig. 1h). Further substantiating a physical and functional interaction between KIF13B and NPHP4, we found that functional invalidation of either KIF13B or NPHP4 caused a similar lack of CAV1 TZ confinement and impaired SMO ciliary accumulation in response to Shh stimulation (this study and ref. 43). The simplest explanation that consolidates these observations is that NPHP4 recruits or tethers KIF13B to the basal body/transition fibres prior to or independently of NPHP4 translocation to the TZ. An elegant study in *Chlamydomonas* showed that once NPHP4 is incorporated at the TZ, it remains stably associated with this compartment^34^. Once KIF13B has been deposited at the basal body and following TZ formation, it is therefore unlikely that KIF13B actively mediates transport of NPHP4 within the ciliary compartment although it cannot be excluded that KIF13B might mediate such transport within other cellular contexts, for example, at cell junctions^52^. Studies using live cell imaging approaches should help to explore these possibilities further.

How might KIF13B control CAV1 tethering at the TZ? Since CAV1 and KIF13B do not co-localize at the ciliary TZ, and because CAV1 accumulated in cilia of KIF13B-depleted cells, KIF13B is unlikely to directly transport CAV1 into cilia and tether it at the TZ. Rather, KIF13B may associate with and deposit one or more factors at the TZ that tether CAV1 to this region as CAV1 diffuses from the periciliary- to the ciliary membrane compartment. The CAV1 TZ tethering factors might include the known KIF13B partners DLG1 (ref. 24) and/or UTRN (ref. 33), which bridge KIF13B with CAV1 (ref. 33), or one or more TZ components yet to be identified. CAV1 likely functions as part of a membrane diffusion barrier in association with further TZ components^31^,^35^,^41^,^53^, limiting ciliary access of SMO in the absence of Shh stimulation. How stimulation with Shh allows SMO to cross this barrier is an open question. SMO has several binding sites for cholesterol, and a recent study using a variety of SMO deletion constructs indicated that cholesterol critically regulates the interaction of the SMO transmembrane domains with the lipid bilayer at the ciliary base^44^. It is possible that CAV1, a cholesterol-binding protein, similarly modulates this interaction and that the ability of CAV1 to organize the TZ lipid bilayer is subject to regulation, for example, by Shh-mediated ciliary exit of PTCH1 that presumably involves recruitment of PTCH1 to CAV1-positive lipid rafts^54^. This scenario is in line with our result showing that purmorphamine-mediated ciliary translocation of SMO is unaffected by KIF13B depletion, implying that KIF13B-mediated effects on SMO ciliary translocation are PTCH1-dependent^46^.

Although defects in ciliary TZ integrity and Shh signalling are hallmarks of many ciliopathies (see above), so far no disease-causing mutations have been identified in KIF13B. However, a recent study using mutant mice in which *Kif13b* was specifically ablated in Schwann cells or oligodendrocytes revealed a requirement for *Kif13b* in peripheral and central nervous system myelination^55^—processes known to involve primary cilia-mediated Shh signalling^56^,^57^,^58^,^59^,^60^. It will therefore be of interest to investigate potential Shh signalling defects in the conditional *Kif13b* mutant mice^55^.

## Methods

### Antibodies

For immunoblot analysis, the following primary antibodies were used (dilutions in parenthesis): rabbit anti-GAPDH (2118, 1:2,000), rabbit anti-phospho Rb^S807/811^ (9308, 1:200) from Cell Signaling Technology; rabbit anti-HA (sc-805, 1:500), rabbit anti-GFP (sc-8334, 1:500), mouse anti-GFP (sc-9996, 1:500) from Santa Cruz; mouse anti-KIF13B #2 (SAB1412812, 1:500), mouse anti-α-tubulin (T5168, 1:5,000), mouse anti-FLAG (F-1804, 1:500) from Sigma; mouse anti-KIF13B #1 (1:500) and rabbit anti-KIF13B (1:500) were generously provided by Athar Chishti, Tufts University. Secondary antibodies used for immunoblot: horseradish peroxidase-conjugated goat anti-mouse (P0447, 1:4,000) or swine anti-rabbit (P0399, 1:4,000) from Dako; alkaline phosphatase-conjugated goat anti-mouse (A1293, 1:5,000) or goat anti-rabbit (A3937, 1:5,000) from Sigma. For IFM analysis, the following primary antibodies were used: rabbit anti-ARL13B (17711-1-AP, 1:1,000), rabbit anti-IFT88 (13967-1-AP, 1:500) from ProteinTech; mouse anti-EEA1 (610456, 1:200), mouse anti-p150^Glued^ (610473, 1:500) from BD Biosciences; rabbit anti-detyrosinated α-tubulin (ab48389, 1:1,000), rabbit anti-SMO (AB7817, 1:200), rabbit anti-PCM1 (ab72443, 1:500), goat anti-Myc (ab9132, 1:1,000) from Abcam; rabbit anti-CAV1 (3238, 1:200), rabbit anti-Myc (2278, 1:1,000) from Cell Signaling Technology; goat anti-PCTN2 (sc-28145, 1:500), mouse anti-SMO (sc-166685, 1:200), rabbit anti-HA (sc-805, 1:250) and rabbit anti-ACIII (sc-588, 1:250) from Santa Cruz; mouse anti-acetylated α-tubulin (T7451, 1:2,000) and rabbit anti-CEP164 (HPA037606, 1:500) from Sigma; rabbit anti-INPP5E (17797-1-AP, 1:250) from Proteintech; rabbit anti-KIF13B (1:500) was a gift from Athar Chishti, Tufts University. Secondary antibodies used for IFM (all from Invitrogen and diluted 1:600, catalogue number in parenthesis): Alexa Fluor 350-conjugated donkey anti-mouse (A-10035) or donkey anti-rabbit (A-10039); Alexa Fluor 488-conjugated donkey anti-mouse (A-21202), donkey anti-rabbit (A-21206) or donkey anti-goat (A-11055), Alexa Fluor 568-conjugated donkey anti-mouse (A-10037), donkey anti-rabbit (A-10042) or donkey anti-goat (A-11057).

### PCR, cloning procedures and plasmids

Primer sequences are listed in Supplementary Table 1. For generation of wild-type mouse *Kif13b* promoter constructs (Supplementary Fig. 1c), relevant regions were PCR amplified from genomic wild-type mouse DNA (gift from Lisbeth B. Møller, Centre for Applied Human Molecular Genetics, Kennedy Centre, Copenhagen, Denmark) and cloned into promoter-less, pGL3-basic Firefly Luciferase vector (Promega) by standard procedures. Mutant versions of the 494 bp *Kif13b* promoter in pGL3 (494 bp wt; Supplementary Fig. 1d) were generated by PCR with relevant mutant primers and cloned into pGL3 by standard procedures. Plasmid encoding human GFP-KIF13B (ref. 24) was provided by Dr Athar Chishti (Tufts University, Sackler School of Graduate Biomedical Sciences, Boston, MA, USA). Plasmids encoding truncated versions of GFP-KIF13B were generated by PCR with relevant primers and GFP-KIF13B plasmid as template, followed by cloning into pEGFP-C1 (Clontech) by standard procedures. Plasmids encoding full-length or truncated human NPHP4 were generated by PCR with relevant primers and human *NPHP4* cDNA as template, followed by cloning into pCMV2-FLAG (Sigma) by standard procedures. Plasmid encoding GFP-KIF13A was generated by PCR with relevant primers and human cDNA as template, followed by cloning into pEGFP-N1 (Clontech). Plasmid encoding FLAG-RPGRIP1L was provided by Dr Sebastian Patzke (Oslo University Hospital, Norway), plasmid encoding GFP-KIF17 was from Dr Geri Kreitzer (Department of Cell & Developmental Biology, Cornell University, NY, USA), GFP-KIF1B plasmid was from Dr Niovi Santama (University of Cyprus, Nicosia, Cyprus), YFP-KIF1A plasmid was from Dr Kristen Verhey (University of Michigan, Ann Arbor, MI, USA), TagRFP-T-RAB8 plasmid was provided by Dr Yuko Mimori-Kiyosue (RIKEN Center for Life Science Technologies (CLST), Kobe, Japan), KIF13B-HA plasmid was from Dr Joel L. Pomerantz (Johns Hopkins University School of Medicine, Baltimore, MD, USA), FLAG-Ap80 plasmid was from Dr Joseph Kissil (Scripps Research Institute, Jupiter, FL, USA), HA-RAB5 plasmids were from Dr Harald Stenmark (Oslo University Hospital, Norway), Renilla Luciferase Reporter vector (pHRG-B) was from Promega, CAV1-mEGFP/FRT/TO (ref. 61) and pSpCas9(BB)-2A-Puro (PX459) (ref. 39) were from Addgene, plasmid encoding HA-tagged Flotillin-2/Reggie-1 was from Dr Claudia Stuermer (Universität Konstanz, Germany), and plasmid encoding Shh-N (ref. 62) was from Drs B.K. Yoder (Deparment of Cell Biology, University of Alabama, USA) and C.J. Haycraft (Department Oral Health Sciences, Charleston, SC, USA). For CRISPR-Cas9 mediated knock out of KIF13B, 20 nt oligos targeting exon 1 in KIF13B (5′-CCGCACCGCCACTTTCACTT-3′) were cloned into pSpCas9(BB)-2A-Puro (PX459) using a published procedure^39^. *Escherichia coli* DH10α was used for transformation and plasmid amplification, and plasmids were purified using NucleoBond Xtra Midi EF Kit from Macherey-Nagel. Plasmid inserts were sequenced at Eurofins MWG Operon, Ebersberg, Germany.

### Transcriptomics and bioinformatics analysis

Transcriptomics analysis of NIH3T3 cells cultured in the presence and absence of serum was performed as described previously^63^. ClustalW2 ( www.ebi.ac.uk/Tools/msa/clustalw2/ 2016) was used for multiple alignment of selected *KIF13B* sequences from different species, CLC workbench 6.7.1.—Limited mode was used for depicting results, and consensus transcription factor binding sites were obtained from the ENCODE consortium using the UCSC Human Genome Browser, February 2009 (GRCh37hg19) Assembly^64^. Computational analysis of the KIF13B amino acid sequence was done using profile-to-profile hidden Markov model (HMM)-HMM searches against the PFAMA database ( http://pfam.sanger.ac.uk) and by HHpred (ref. 28) as performed previously^65^. The conserved C2 domains in human KIF13B were identified by using amino acid 861-1,000 as a search query in PSI-BLAST searches against the non-redundant database (nr). A minimum of three reiterative searches were used to construct a multiple sequence alignment (MSA) suitable for HMM-based searches against the PFAM database. High sequence homology to the RPGRIP1N-C2 type domain (C2-C2_1 domain; registered as DUF3250 in the PFAM database) was readily attained producing highly significant expect values in the range of E=1e-07. No other type of C2 domain family matched the KIF13B query with high probability indicating that the KIF13B C2 domain belongs to the RPGRIP1N-C2/C2_1 domain family. Indeed, database searches for homologous sequences of the KIF13B/KLP-6 C2 domain against the human proteome (using the KLP-6 RPGRIP1N-C2 domain between residues 701-824 as query) identified, besides the kinesin-3 members KIF1A, KIF1B, KIF13A and KIF13B, RPGRIP1 and RPGRIP1L as the other highly significant matches (RPGRIP1 E-value=1.6e-05 and RPGRIP1L E-value=2.4e-06). Likewise, a reciprocal HMM-HMM search seeded with the RPGRIP1N-C2/C2-C2_1 domains of either RPGRIP1 or RPGRIP1L found *C. elegans* KLP-6 as the highest scoring match besides RPGRIP1 and RPGRIP1L themselves. A second iterative search using a query constructed from the initial MSA merged with the matched KLP-6 C2 domain, identified all the above kinesin-3 members as well as to the C2 domains of the centrosome/cilia proteins C2CD3 and more remotely to NPHP4. Similar searches seeded with divergent C2 family domains failed to identify any homologues of KIF13B, thus confirming that KIF13B is a genuine RPGRIP1/1L family member. Multiple sequence alignment was obtained and edited in Jalview^66^, the consensus calculated and coloured using ClustalX, as implemented in Jalview. Construction of cladograms was also performed in Jalview. Homology modelling of 3D structures was done with Modeller^67^. Analysis of resulting 3D model coordinates was done using Discovery Studio 3.5 Visualizer.

### Cell culture and transfections

Swiss NIH3T3 mouse fibroblasts (laboratory stock, originally derived from American Type Culture Collection (ATCC) clone CRL-1658) were grown at 37 °C in Dulbecco's modified Eagle's medium (DMEM, Gibco) with 10% heat inactivated fetal bovine serum (FBS, Gibco) and 10 ml l^**−**1^ penicillin-streptomycin (Gibco) using 5% CO_2_ and 95% humidity. MEFs were isolated using Freshney's protocols 11.1 and 11.5 (ref. 68) and cultured at 37 °C in 45% DMEM and 45% F12+L-glutamine (InVitrogen) supplemented with 10% heat inactivated FBS and 10 ml l^**−**1^ penicillin-streptomycin, using 5% CO_2_ and 95% humidity. The *Dynll1*^GT/GT^ MEFs were kindly provided by Dr Dominic Norris (MRC Harwell, Oxfordshire, UK) and were described recently^40^. Generation and characterization of *Nphp4*^−/−^ MEFs will be described elsewhere (Bizet *et al*., in preparation). Microscopy studies using MEFs were performed with cells cultured only in low passage. NTERA-2 (NT2) cells (ATCC CRL-1973) and HEK293T cells (ATCC CRL-3216) were cultured at 37 °C in DMEM with 10% heat-inactivated FBS and 10 ml l^**−**1^ penicillin-streptomycin. Neuronal differentiation of NT2 cells was induced by treatment with 10 μM retinoic acid for 21 days; retinoic acid-containing medium was changed every 3 days. The RPE1 cells (laboratory stock, derived from the immortalized hTERT RPE1 cell line, ATCC CRL-4000) and were grown in 45% DMEM and 45% F-12 (Ham; Sigma) with 10% FBS and 10 ml l^**−**1^ penicillin-streptomycin; cultures were passaged every 3-4 days. Generation of KIF13B knock out RPE1 cell lines was done using CRISPR-Cas9 methodology^39^ by utilizing plasmid pSpCas9(BB)-2A-Puro (PX459) encoding KIF13B-specific guide RNA targeting the first exon of KIF13B (5′-CCGCACCGCCACTTTCACTT-3′). Sequencing of PCR-amplified genomic DNA from one of these cell lines (clone #3 used for functional analyses) revealed a homozygous frameshift mutation in exon 1 of *KIF13B* (single nucleotide insertion in codon 9; GCG→GCTG). For transfection of NIH3T3 cells, cells were grown to 70% confluency in 9.6 cm^2^ petri dishes, before transfection. Transfections were carried out using DharmaFECT Duo (Dharmacon) according to manufacturer's instructions. Twenty-four hours after transfection, cells were harvested or the medium was changed to serum-free medium and cells incubated for additional 24 h before harvest.

For KIF13B protein depletion, RPE1 cells were transfected with siRNA two times over two days and incubated additionally two days. To obtain growth arrest prior to knockdown (to avoid biased outcome due to cell cycle effects), cells were grown to approximately 80% confluence in 9.6 cm^2^ petri dishes before first transfection; 5-6 h after transfection the medium was changed. Two previously validated KIF13B-specific siRNAs were used, both purchased at Eurofins MWG Operon: siKIF13B-1 (5′-CCGAAGGUGUUUGCUUAUGAU-3′) corresponding to shRNA2b in ref. 26, and siKIF13B-2 (5′-GUGCCUUGGAGAGAAUAUC-3′) described in ref. 69. In addition, KIF13B-specific esiRNA (SigmaMISSION esiRNA human KIF13B, cat. No. EHU088721) was used in the experiment in Supplementary Fig. 6a. Ctrl siRNA (5′-UAAUGU AUUGGAAUGCAUA(dTdT)-3′ was from Eurofins MWG Operon. A previously validated human CAV1-specific siRNA was used^70^: siCAV1 (5′-AAGAGCUUCCUGAUUGAGA-3′). All transfections with siRNA (final concentration 250 nM) were carried out using DharmaFECT Duo as described above for NIH3T3 cells. Six hours after transfection fresh growth medium was added. The cells were subjected to double transfection with an interval of 24 h before 24 h of serum deprivation prior to further manipulation or analysis. For transfection of RPE1 cells with plasmids for live cell imaging, cells were grown in six well-plates to a confluence of ca. 90%. In total, 1 μg of plasmid (GFP-KIF13B and TagRFP-T-RAB8) was transfected using FuGene 6 (E2692, Promega) as transfection reagent and cells were incubated for 24 h followed by 20 h of serum deprivation.

For plasmid transfection of HEK293T cells, 8 μg DNA was transfected into cells in a 15 cm dish using FuGene 6 and incubated additionally 48 h. For IFM of RPE1 cells 1 μg DNA was transfected into the cells using FuGene 6. Cells were transfected 2 h before change to serum-depleted medium for no more than 16 h prior to fixing. For CAV1-GFP and GFP-FLOT2 localization studies, no more than 100 ng plasmid was tranfected into cells.

### Rapid amplification of cDNA 5′ ends (5′RACE)

The 5′/3′ RACE Kit, 2nd Generation (Roche Applied Science) was used to amplify the untranslated region of human and mouse *KIF13B*, following the instructions of the manufacturer. For purification of cDNA, the GeneJet PCR purification Kit (Fermentas) was used. RNA was collected from human differentiated NT2 cells and MEFs serum-deprived for 24 h. Amplification products were sequenced by Eurofins MWG Operon. For Dual Luciferase reporter assay, NIH3T3 cells were grown and transfected with pGL3-based plasmids and pHRG-B as described above. Cells were lysed in Passive Lysis Buffer (5 × PLB) from the Dual-Luciferase Reporter Assay System Kit (Promega), the lysates collected in eppendorf tubes and stored at −20 °C. Samples were thawed and luciferase activity measured on a RamCon Fluostar Optima plate reader following the manufacturer's instructions. Briefly, 20 μl of each lysate was dispensed in a white 96-well Polystyrene Cell Culture Microplate (Greiner bio-one). One hundred microlitres LAR II was added and mixed by careful resuspension, and the Firefly Luciferase activity was measured. One hundred microlitres Stop & Glo Reagent was then added and mixed by careful resuspension, and Renilla Luciferase activity was measured. The Firefly Luciferase activity was normalized to that of the Renilla Luciferase.

### Ligand stimulation assays, SDS-PAGE, immunoblotting

For rescue of ciliary length with WNT5A, RPE1 cells were treated with PBS or 200 ng ml^−1^ WNT5A (R&D Systems) for 6 h prior to IFM. For Shh stimulation assays, Shh-N CM was generated as previously described^62^. Cells were cultured in a 1:1 mixture of conditioned medium and DMEM prior to harvest or fixation. Stimulation of serum-starved, siRNA treated RPE1 cells with purmorphamine (1 μM; Sigma) was done for 12 h prior to IFM analysis. Methyl-β-cyclodextrin (MBCD; 5 mM final concentration) cholesterol depletion assay was performed on serum-starved RPE1 cells with or without Shh-N CM stimulation. Cells were incubated in fresh serum-free medium 1 h prior to MBCD addition, incubated for 1 h and subsequently fixed for IFM analysis as described below.

Analysis by SDS-PAGE and immunoblotting with relevant antibodies was performed using the Novex system from Invitrogen and by following the protocol supplied by the vendor. Blots were incubated in primary antibodies at appropriate dilutions, incubated with relevant horse radish peroxidase-conjugated secondary antibodies, and developed with FUSION-Fx chemiluminescence system from Vilber Lourmat. Images were processed in Adobe Photoshop CS6. Quantification of immunoblot signals was done using ImageJ software. Original scans of all immunoblots are provided in Supplementary Figs 7–12.

### RNA isolation, RT-qPCR analysis and statistical analyses

Total RNA was extracted from cells using Nucleospin RNA II Kit (Machery-Nagel) according to the manufacturer's instructions. One microgram RNA was reverse transcribed into cDNA using SuperScript III (Invitrogen), following the provided instructions. Relative qPCR was carried out in 20 μl reactions using SYBR *premix Ex Taq* (TaKaRa). Nineteen microlitres mastermix was mixed with 1 μl cDNA and each reaction was measured in triplicates on the Stratagene Mx4000 Multiplex Quantitative PCR system. The relative amount of mRNA normalized to beta-2microglobulin (B2M) was calculated using the Pfaffl method. Statistical analyses were performed using GraphPad Prism 6 software.

### Immunofluorescence microscopy and live imaging analysis

Unless stated otherwise, IFM analysis was carried out as follows. Cells grown on glass coverslips were washed once in ice cold PBS, fixed with 4% paraformaldehyde (PFA) solution, permeabilized with permeabilization buffer (PBS with 0.1% (v/v) Triton-X100 and 1% (w/v) bovine serum albumin (BSA)) and subjected to IFM as described previously^21^. For IFM analysis of KIF13B (endogenous and tagged) or other kinesin-3 motors, cells were treated the same way except that they were subjected to a brief pre-extraction with permeabilization buffer containing either 1 μM AMP-PNP prior to fixation with PFA or with gentle incubation with ice cold CSK pre-extraction buffer (10 mM Hepes, pH 7.0, 100 mM NaCl, 300 mM sucrose and 10 mM EDTA) containing 0.5% Triton X-100) for 5 min on ice prior to fixation with ice cold 4% PFA. After fixing cells were allowed to air dry to prevent further cell loss from the coverslips. Imaging was done using a motorized Olympus BX63 upright microscope with a DP72 colour, 12.8 megapixel, 4,140 × 3,096 resolution camera and differential interference contrast. The software used was Olympus CellSens dimension, which was able to do 3D isosurfacing on captured z stacks, and images were processed for publication using Adobe Photoshop CS4 version 11.0. For quantification of ciliary SMO levels, an outline was drawn around each cilium and using the measurement and region of interest (ROI)-function in the CellSens dimension software (Olympus) the Mean Green Fluorescence Intensity was measured in this area along with a background reading. The corrected mean fluorescence in the cilia was calculated by subtracting the corresponding background value. For live imaging analysis (Supplementary Fig. 1e), RPE1 cells were grown in six-well plates and transfected as described previously. Prior to imaging the cover slip was placed in the specimen chamber, 2 ml of fresh pre-heated medium was added and the chamber installed in the incubator connected to the microscope, keeping the temperature of the chamber at 37 °C during the whole imaging process. Imaging of the red and green channel was performed simultaneously on a Spinning disk confocal microscope, as described previously^71^.

### Immunoprecipitation and size exclusion chromatography

HEK293T cells were transfected the day before IP or gel filtration assays. Cells were harvested in ice cold, modified EBC buffer (500 mM NaCl, 10 mM Tris-HCl, 0.5% NP-40 and protease inhibitor cocktail (Roche)). For FLAG and GFP IP experiments, cleared cell extracts were incubated 1 h with either 20 μl Anti-FLAG (M2) conjugated beads (Sigma, catalogue number A2220) or protein G-conjugated sepharose beads (GE Healthcare, catalogue number 17-0618-01) bound to rabbit anti-GFP antibody (Santa Cruz, catalogue number sc-8334) under constant rotation (4 °C). Immunocomplexes were washed five times before elution with 3 × FLAG peptide (Sigma, catalogue number F4799) for FLAG IP or with SDS-PAGE sample buffer (GFP IP). Eluted FLAG-proteins complexes were purified further by micropore filter centrifugation. For gel filtration assay cleared HEK293 cell extracts were run on a NaPO_4_ buffered Superose 6 column powered by a HPLC pump to resolve protein complexes. Fractions of 500 μl were collected and concentrated by speedvac centrifugation. IP with bacterially purified GST-NPHP4-C2^650-839^ (500 ng μl^−1^) with high salt-purified GFP- KIF13B-C2^861-1,000^ was done by washing GFP-KIF13B-C2^861-1,000^-anti-GFP immunocomplexes two times in a high salt buffer (600 mM NaCl, 10 mM Tris-HCl, 0.1% NP-40 and protease inhibitor cocktail (Roche)) before adding GST-NPHP4-C2^650-839^ in modified EBC buffer (140 mM NaCl, 10 mM Tris-HCl, 5 mM EDTA, 0.5% NP-40 and protease inhibitor cocktail (Roche)) and subsequent standard GFP IP.

### Data availability

The data that support the findings of the current study are available from the corresponding author on reasonable request.

## Additional information

**How to cite this article:** Schou, K. B. *et al*. KIF13B establishes a CAV1-enriched microdomain at the ciliary transition zone to promote Sonic hedgehog signalling. *Nat. Commun.* **8,** 14177 doi: 10.1038/ncomms14177 (2017).

**Publisher's note:** Springer Nature remains neutral with regard to jurisdictional claims in published maps and institutional affiliations.

## Supplementary Material

Supplementary Information Supplementary Figures, Supplementary Table 1, and Supplementary References
