# Supplementary material for: KIF13B establishes a CAV1-enriched microdomain at the ciliary transition zone to promote Sonic hedgehog signalling
Source: Nat Commun. 2017 Jan 30;8:14177. doi: 10.1038/ncomms14177 (PMC5290278; doi:10.1038/ncomms14177)
Supplement: Supplementary Information — Supplementary Figures, Supplementary Table 1, and Supplementary References [file ncomms14177-s1.pdf]

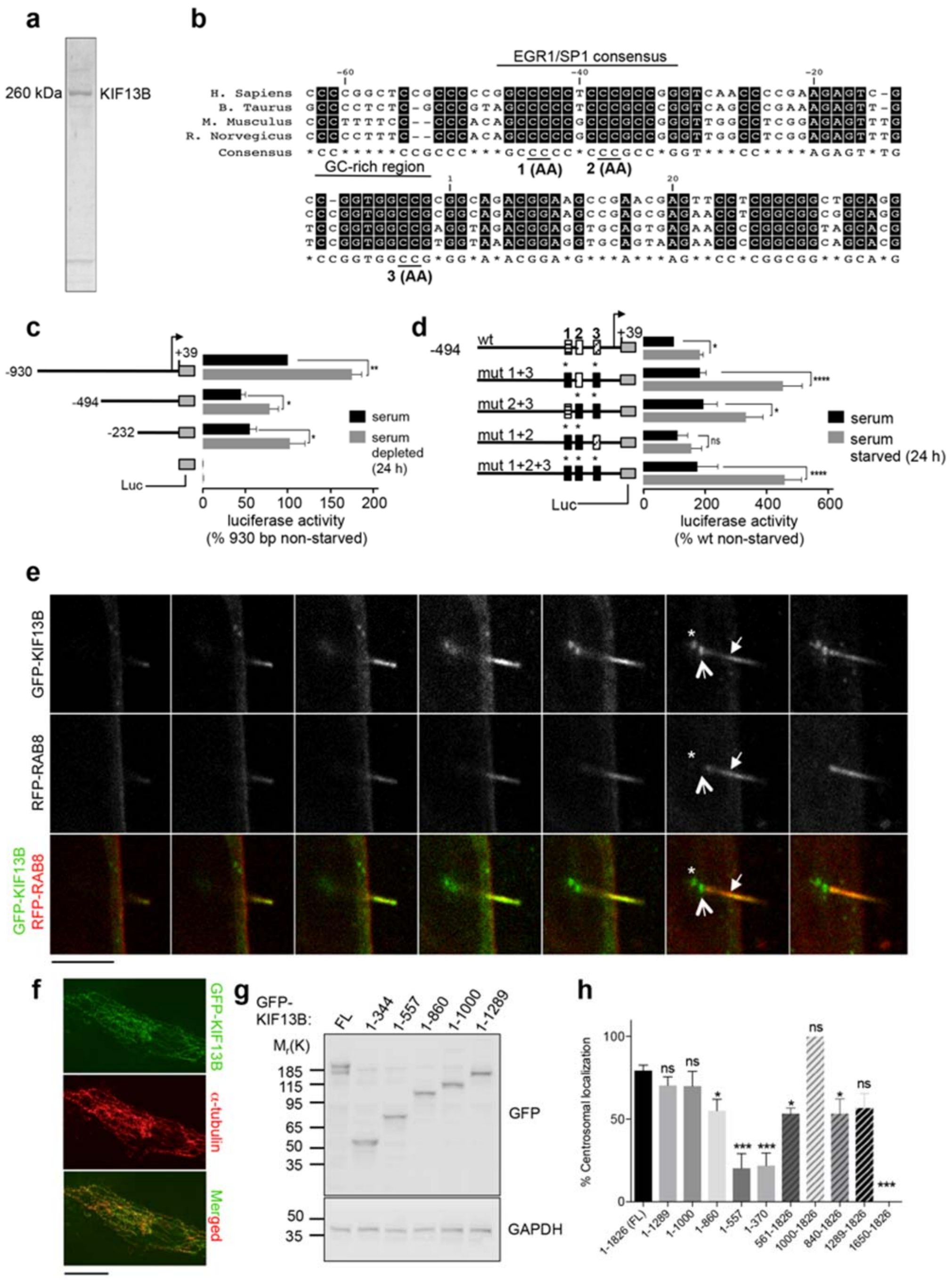

Supplementary Figure 1. *Kif13b* expression and localization in serum starved cells. (**a**) Immunoblot of NIH3T3 cell lysates after 24 hrs of serum deprivation, using rabbit polyclonal KIF13B antiserum. (**b**) Multiple alignment *KIF13B* promoters from various species. The transcription start site (TSS, +1) was determined by 5'RACE in NT2 and MEFs. Conserved regions (underlined) include consensus binding sites for

EGR1, SP1, and a GC-rich region. Results generated by the Encyclopedia of DNA Elements (ENCODE) project confirmed that EGR1 and SP1 bind to the *KIF13B* promoter [1]. The position of mutated residues in different 494 bp *Kif13b* promoter constructs used in luciferase assays (panel c) is shown. (**c**) Luciferase assay in NIH3T3 cells of *Kif13b* promoter constructs. Results (n=3) are shown relative to the 930 bp construct, non-starved. Bars: mean ± s.e.m. P values result from one- (control) or two-tailed t-test. (**d**) Luciferase assay (NIH3T3 cells) of *Kif13b* wild type (wt) and mutant (mut) promoter constructs. Results (n=3) are expressed relative to the wt 494 bp construct, non-starved. Bars: mean ± s.e.m. P values result from two-way ANOVA followed by Sidak's multiple comparison (****, $P \leq 0.0001$; **, $P \leq 0.01$; *, $P \leq 0.05$; ns, $P \geq 0.05$, not significant). (**e**) Live imaging of RPE1 cells expressing GFP-KIF13B and TagRFP-T-RAB8, serum-starved 20 hrs. Upper panel: localization of GFP-KIF13B along (closed arrow) or at the base (open arrow) of the cilium, and to the centrosome (asterisk). Middle panel: cilia marker, TagRFP-T-RAB8. The montage shows frame 7-13 from a z-stack created using a spinning disk confocal microscope, 100 ms. (**f**) IFM of detergent extracted RPE1 cells expressing GFP-KIF13B (green). Microtubules were stained with α-tubulin antibody (red). (**g**) Immunoblot of RPE1 cells expressing various GFP-KIF13B fusions. (**h**) Quantification of centrosomal localization of GFP-KIF13B fusions (non-ciliated RPE1 cells; analysis of motor-less fusions was done on detergent extracted cells. Data for FL were identical with or without detergent). Bars: mean ± s.e.m. P values result from one-way ANOVA followed by Dunnett's multiple comparison test (***, $P \leq 0.001$; *, $P \leq 0.05$; ns, $P \geq 0.05$, not significant). 10-30 cells analyzed per condition (n=3, except for 1-1826 (FL) n=6). Scale bars: 5 μm in **e**; 10 μm in **f**.

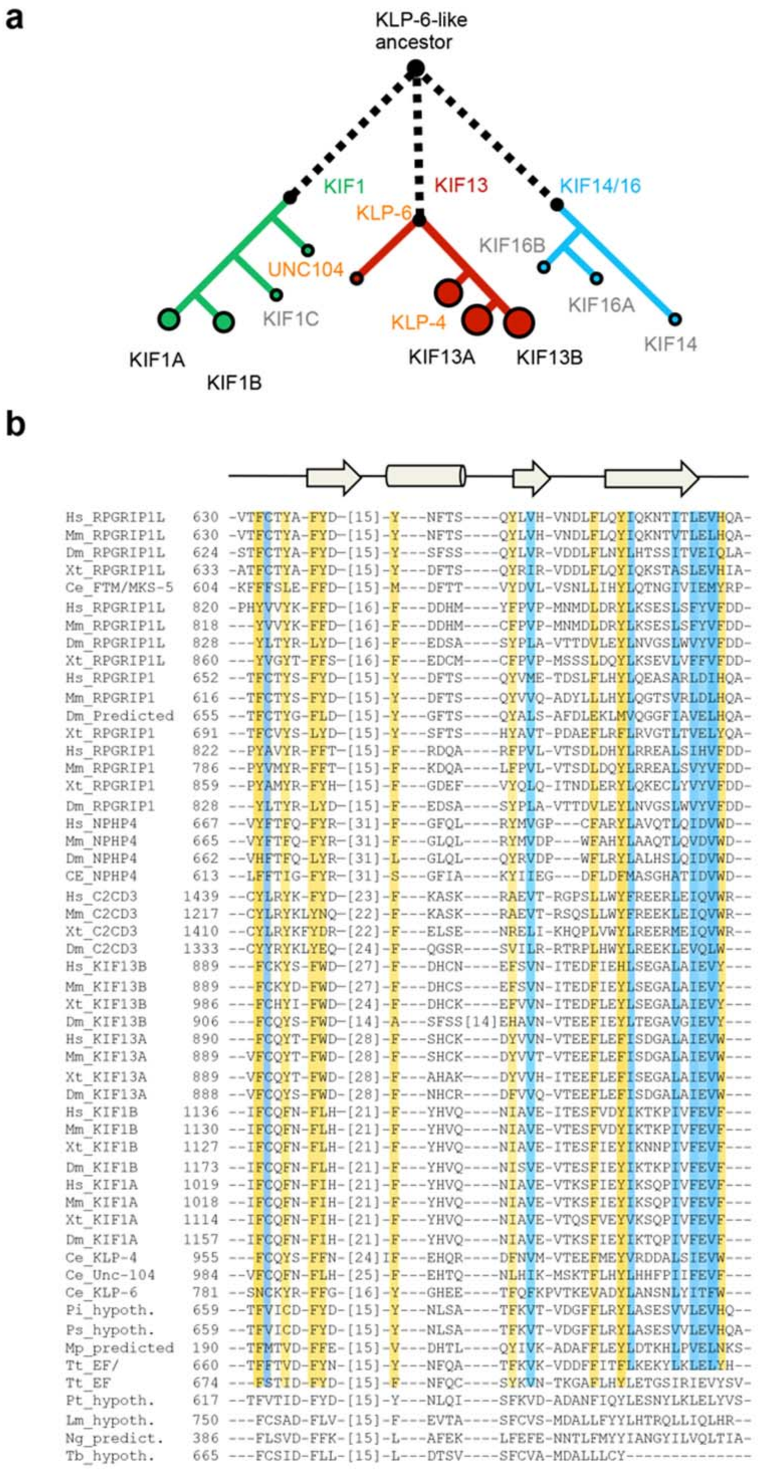

Supplementary Figure 2. Defining the aromatic profile of the RPGRIP1N-C2 domain. (a) Schematic diagram showing evolutionary relationship between kinesin-3 family members. Black and orange font represent human and *C. elegans* kinesins,

respectively. Circle size indicates sequence homology to the KLP-6 predecessor. (**b**) Expanded alignment of selected RPGRIP1N-C2 type domains from different organisms identified by HHpred searches. The defining signature of aromatic amino acids specific to the RPGRIP1-type C2 domains are highlighted in orange. Homologous residues other than those found in aromatic amino acid profiles are shown in blue. The predicted secondary structure interpretation is shown above the alignment. Cylinders: α-helix; arrows: β-sheets. Hs, *Homo sapiens*; Mm, *Mus musculus*; Dr, *Drosophila melanogaster*; Xt, *Xenopus tropicalis*; Ce, *Caenorhabditis elegans*; Pi, *Phytophthora infestans*; Ps, *Phytophthora sojae*; Mp, *Micromonas pusilla*; Tt, *Tetrahymena thermophila*; Pt, *Paramecium tetraurelia*; Lm, *Leishmania major*; Ng, *Naegleria gruberi*, and Tb, *Trypanosoma brucei.*

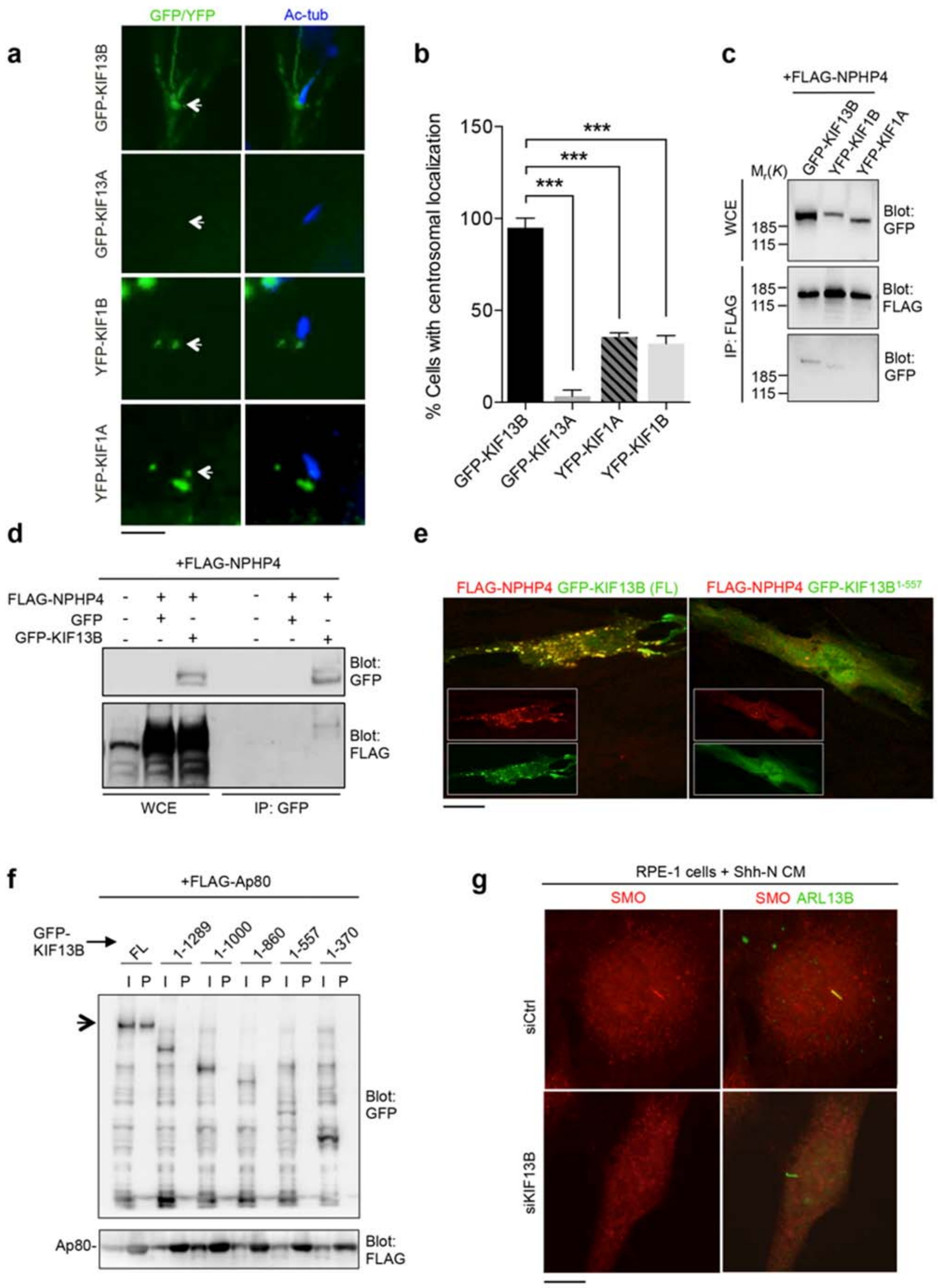

Supplementary Figure 3. Kinesin-3 localization and KIF13B-NPHP4 binding specificity. (a) IFM of detergent extracted RPE1 cells expressing the indicated fusion proteins in green. The cilium was marked with Ac-tub antibody (blue, arrowhead). (**b**) Quantification of centrosome localization of indicated fusion proteins, based on data

as shown in (a). Bars: mean ± s.e.m. P values result from one-way ANOVA followed by Dunnett's multiple comparisons test (***, P≤0.001). At least 10 cells were analyzed per condition (n=3). (**c**) FLAG IP of HEK293T cells co-expressing FLAG-NPHP4 and GFP- or YFP-tagged kinesin-3 motors. Samples were analysed by immunoblot with indicated antibodies. WCE: whole cell extract. (**d**) GFP IP of HEK293T cells co-expressing FLAG-NPHP4 and GFP (control) or GFP-KIF13B. Samples were analysed by immunoblot with indicated antibodies. (**e**) IFM of RPE1 cells co-expressing FLAG-NPHP4 (red) and FL GFP-KIF13B (left panels) or GFP-KIF13B (1-557) lacking the NPHP4-binding C2 domain region (right panels). (**f**) Immunoblot of a control IP experiment in which HEK293T cells co-expressing FLAG-tagged Angiomotin p80 (FLAG-Ap80) and various GFP-KIF13B fusions were subjected to IP with FLAG antibody. Input (I) and pellet (P) fractions were analysed with indicated antibodies. FLAG-Ap80 co-precipitates with GFP-KIF13B (FL; arrow) but not with truncated GFP-KIF13B fusions. (**g**) IFM of ciliary SMO staining (red) in control siRNA treated (siCtrl) and KIF13B siRNA-depleted (siKIF13B) RPE1 cells, stimulated with Shh-N CM. The cilium was stained with antibody against ARL13B (green) and the SMO antibody used was from Santa Cruz. Scale bars: 5 µm in **a**; 10 µm in **e**, **g**.

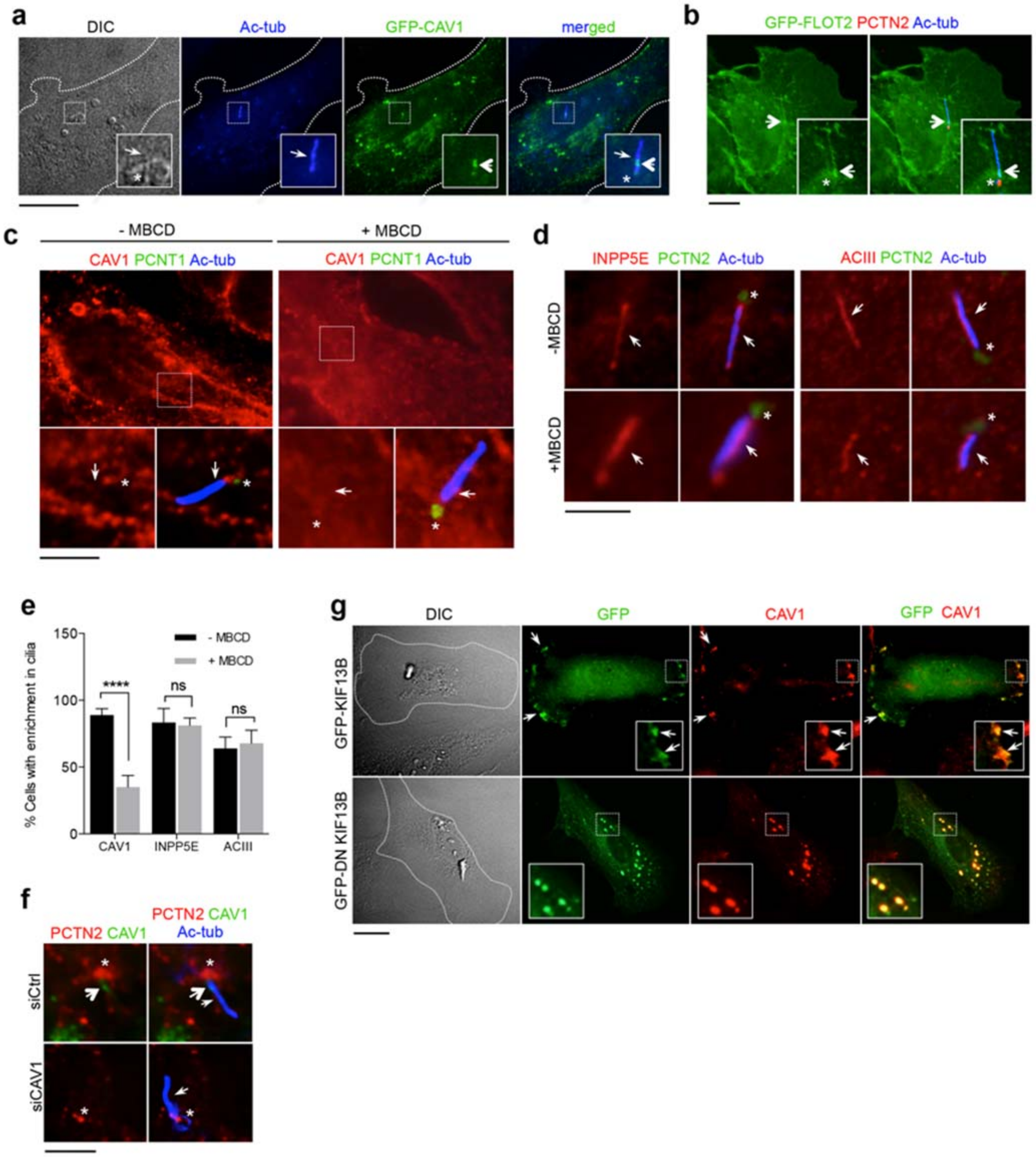

Supplementary Figure 4. Analysis of CAV1 and Flotillin-2 localization. (**a, b**) IFM of ciliated RPE1 cells expressing GFP-CAV1 or GFP-FLOT2, using antibodies as indicated. Insets: magnified images of the cilium (closed arrow) and centrosome (asterisk) with the TZ marked with an open arrow. **(c, d, f)** IFM of RPE1 cells subjected to MBCD treatment (**c, d**) or CAV1 siRNA depletion (**f**) prior to PFA fixation and staining with indicated antibodies. Closed arrow, cilium; open arrow, TZ; asterisk, centrosome. (**e**) Quantification of data in (**c, d**). 30-80 cells analyzed per condition (n=3). Bars: mean ± s.e.m. P values result from two-way ANOVA followed

by Sidak's multiple comparisons test (****, $P \leq 0.0001$; ns, $P \geq 0.05$, not significant). (**g**) IFM of cells similar to those shown in (Fig. 4k), but with focus on cell edges. Closed arrows: co-staining of CAV1 with GFP-KIF13B at cellular protrusions (upper panels) and with GFP-DN KIF13B in endosomal vesicles (lower panels). Insets: enlargement of the boxed regions. Scale bars: 10 μm in **a**, **c**; 5 μm in **b**, **d**, **f**; 20 μm in **g**.

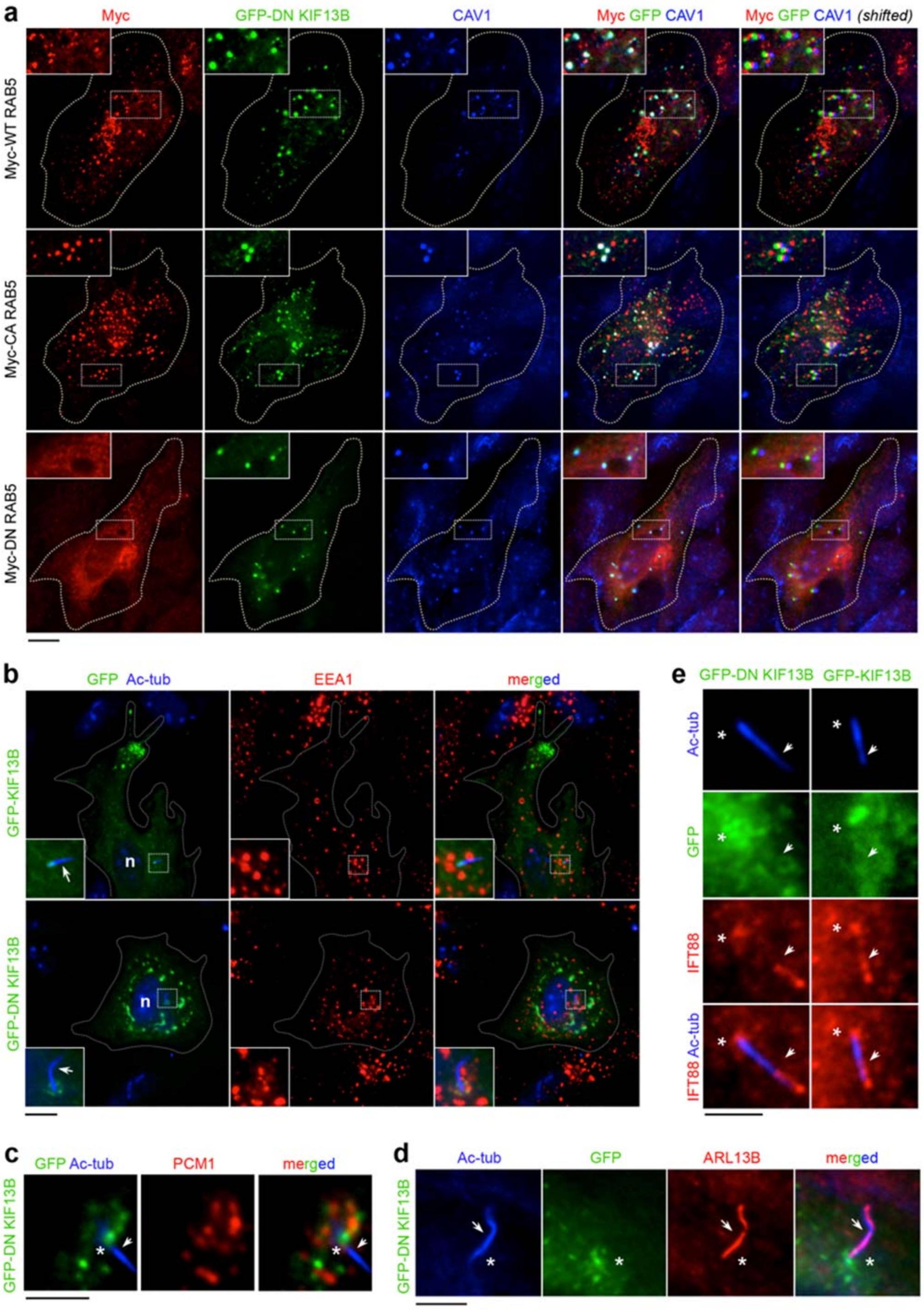

Supplementary Figure 5. IFM in RPE1 cells expressing GFP-KIF13B or GFP-DN KIF13B. (**a**) IFM of RPE1 cells co-expressing GFP-DN KIF13B (green) and Myc-RAB5 wild type (WT), Myc-RAB5^{Q79L} (constitutively active; CA), or Myc-RAB5^{S34N} (dominant negative; DN), as indicated. Cells were fixed with PFA and

stained with antibodies against Myc (red) and CAV1 (blue). Quantitative analysis revealed that in cells expressing Myc-WT RAB5, 79% of the CAV1+GFP-DN KIF13B-positive puncta were positive for Myc-RAB5 (n=80), whereas for Myc-RAB5^{Q79L} or Myc-RAB5^{S34N} expressing cells the percent co-localization was 75% (n=84) and 15% (n=98), respectively, in three (Myc-WT RAB5 and Myc-RAB5^{Q79L}) or two (Myc-RAB5 RAB5^{S34N}) independent experiments. **(b-e)** Ciliated RPE1 cells expressing GFP-KIF13B or GFP-DN KIF13B (green) were stained with antibody against Ac-tub (blue) to mark the ciliary axoneme as well as antibodies (red) specific for EEA1 (**b**), PCM1 (**c**), ARL13B (**d**), and IFT88 (**e**). Insets: enlargements of the boxed regions. Arrows, primary cilium; asterisks, ciliary base; n, nucleus. Scale bars: 10 µm in **a, b**; 5 µm in **c-e**.

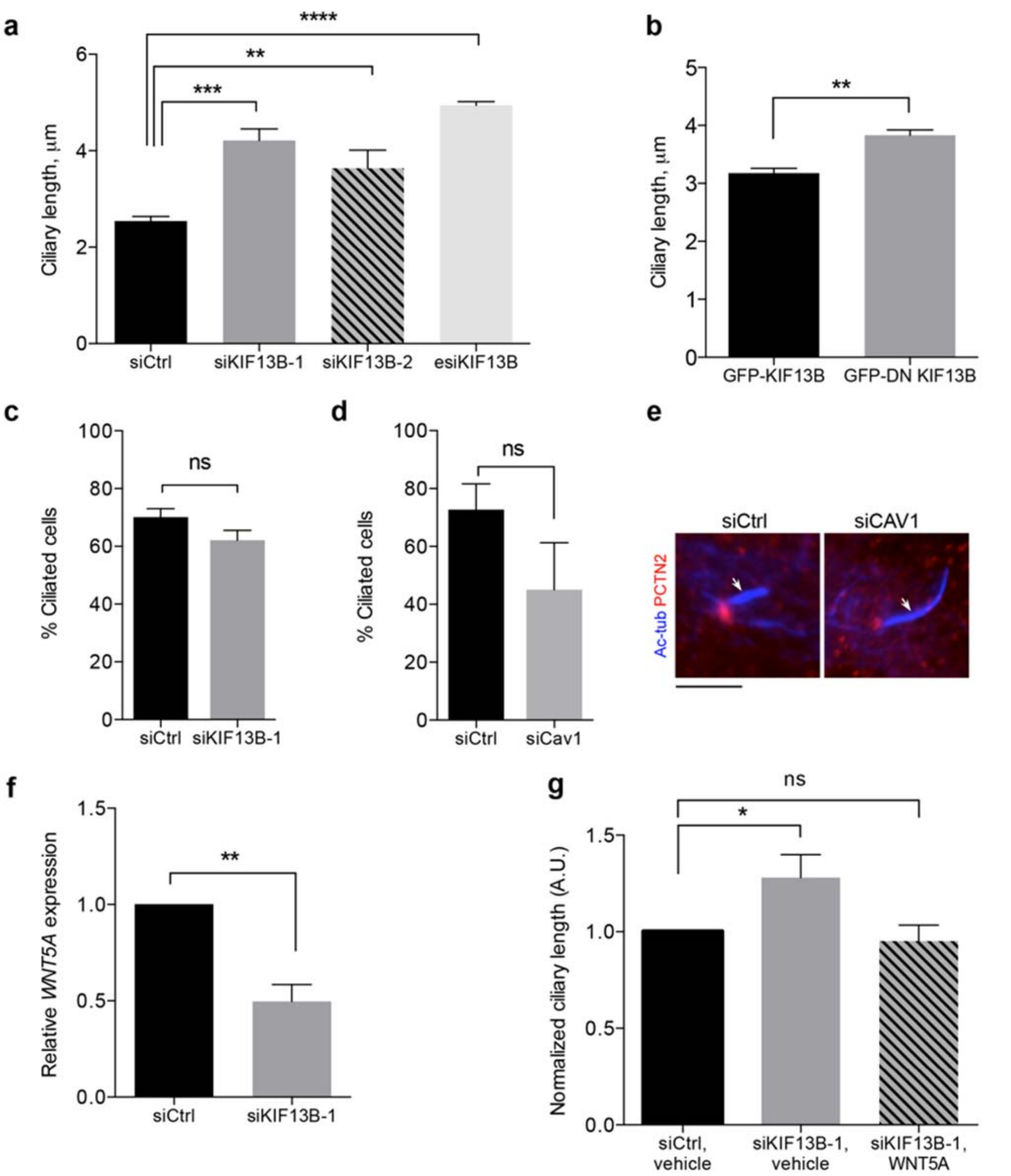

Supplementary Figure 6. KIF13B regulates ciliary length and WNT5A expression. (a) KIF13B depletion causes cilium elongation. RPE1 cells were depleted for KIF13B using three different siRNAs, cells serum-starved for 48 hrs, fixed with PFA and subjected to IFM using Ac-tub antibody to label cilia. Ciliary length was measured for at least 100 cells experiment (n=3). (**b**) Ciliary length in GFP-DN KIF13B or GFP-KIF13B expressing RPE1 cells. Cilia were visualized by IFM with Ac-tub antibody and at least 120 cilia measured per condition (n=3). Bars in (**a**, **b**)

represent mean ± s.e.m. P values result from one-way ANOVA (**a**) and students t-test (**b**) (****, P≤0.0001; ***, P≤0.001; **, P≤0.01). (**c**) Ciliation frequency in control or KIF13B siRNA-treated, serum-starved RPE1 cells (n=3). Approximately 500 cells analyzed per conditon. (**d**) Ciliation frequency in serum-starved RPE1 cells treated with CAV1 siRNA. Fifty cells analyzed per condition (n=3). Bars in (**c, d**) represent mean ± s.e.m. P values result from two-tailed t-test (ns, P≥0.05, not significant). (**e**) IFM of control and CAV1 siRNA-treated, serum-starved RPE1 cells. Cells were stained with antibody against PCTN2 (red) and Ac-tub (blue), as indicated. Arrows, cilium. (**f**) Reduced *WNT5A* expression in KIF13B-depleted cells, determined by RT-qPCR analysis. The analysis was performed on serum-starved RPE1 cells and *B2M* was used as reference gene (n=3). (**g**) Addition of WNT5A to KIF13B-depleted RPE1 cells rescues the ciliary length phenotype. Ciliary length was measured in KIF13B-depleted and control-siRNA transfected RPE1 cells treated with vehicle (PBS) or WNT5A (200 ng ml^{-1}) for 6 hrs (n=3, measuring 50-150 cilia per condition per experiment). Bars in (**f**, **g**) represent mean ± s.e.m . P values result from students t-test (P≤0.001; **, P≤0.01; *, P≤0.05; ns, P≥0.05, not significant). Scale bar in **e**: 5 µm.

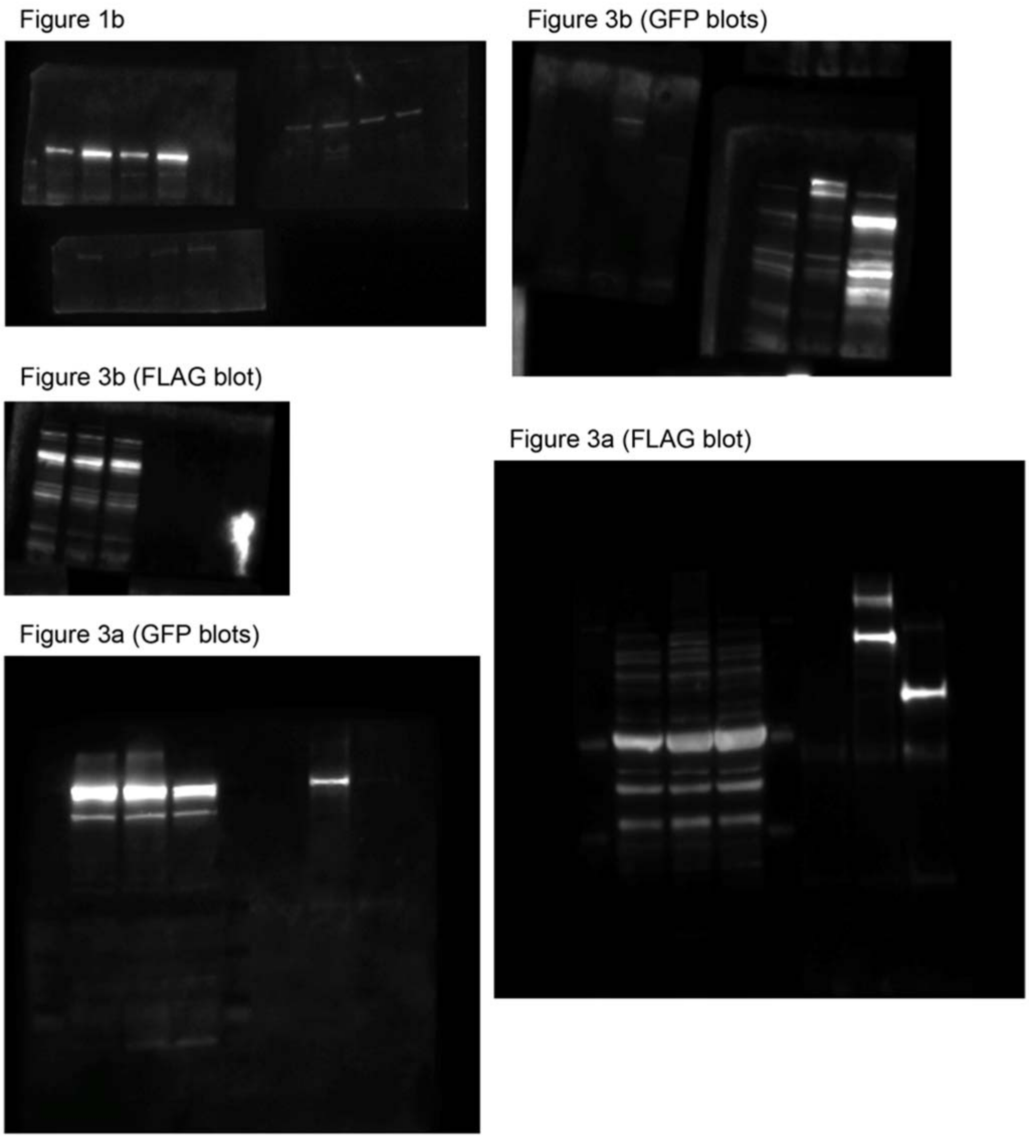

Supplementary Figure 7. Original blots for Figure 1b, Figure 3a and Figure 3b.

Figure 3c (upper panel: HA blot; lower panel: FLAG blot))

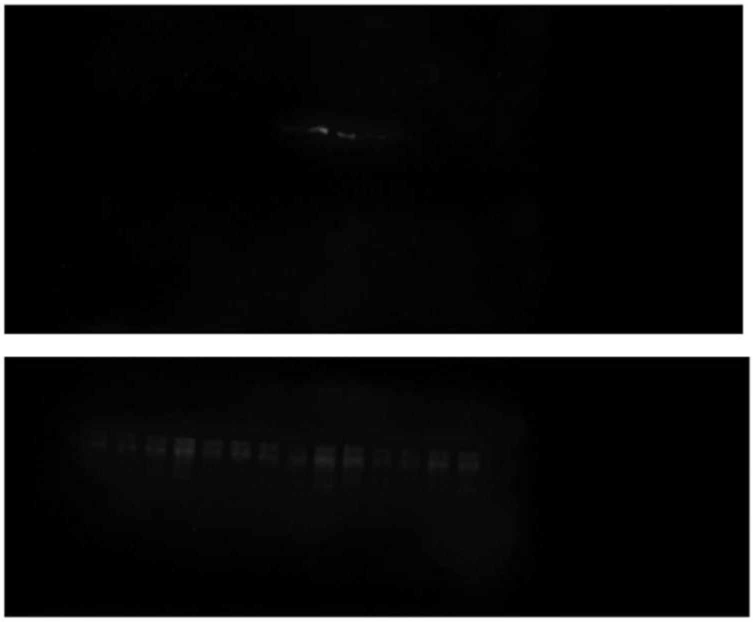

Figure 3d (FLAG blots)

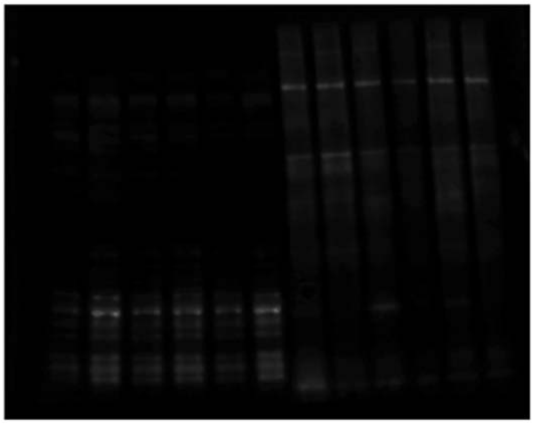

Figure 3d (GFP blots)

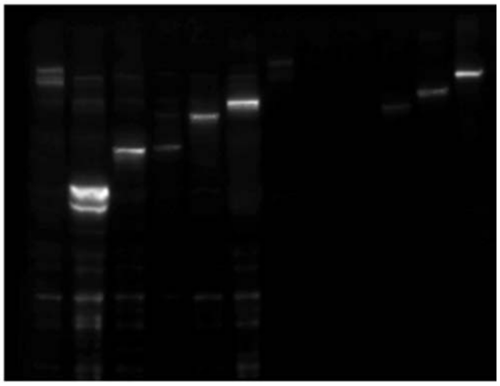

Supplementary Figure 8. Original blots for Figure 3c and Figure 3d.

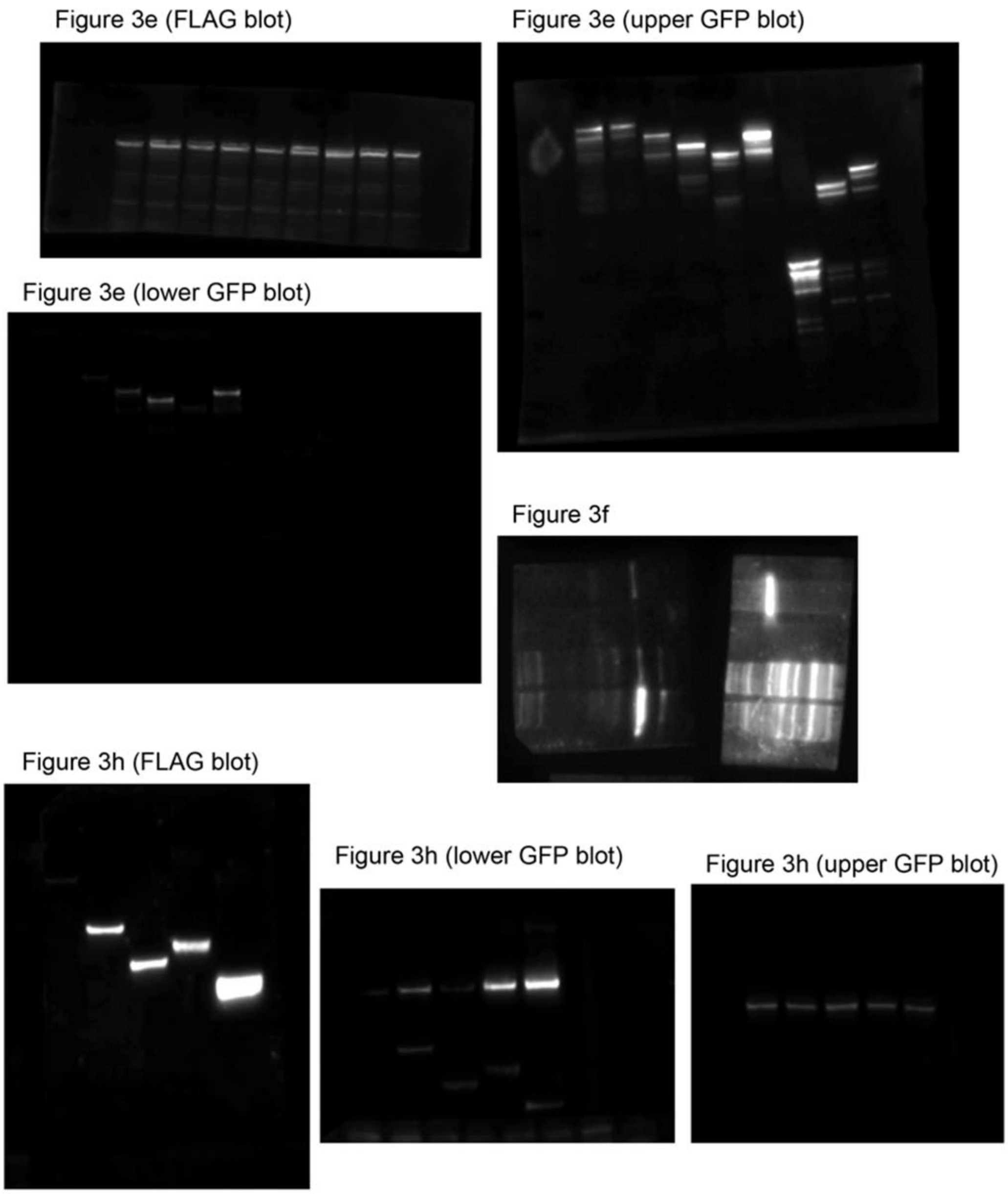

Supplementary Figure 9. Original blots for Figure 3e, Figure 3f and Figure 3h.

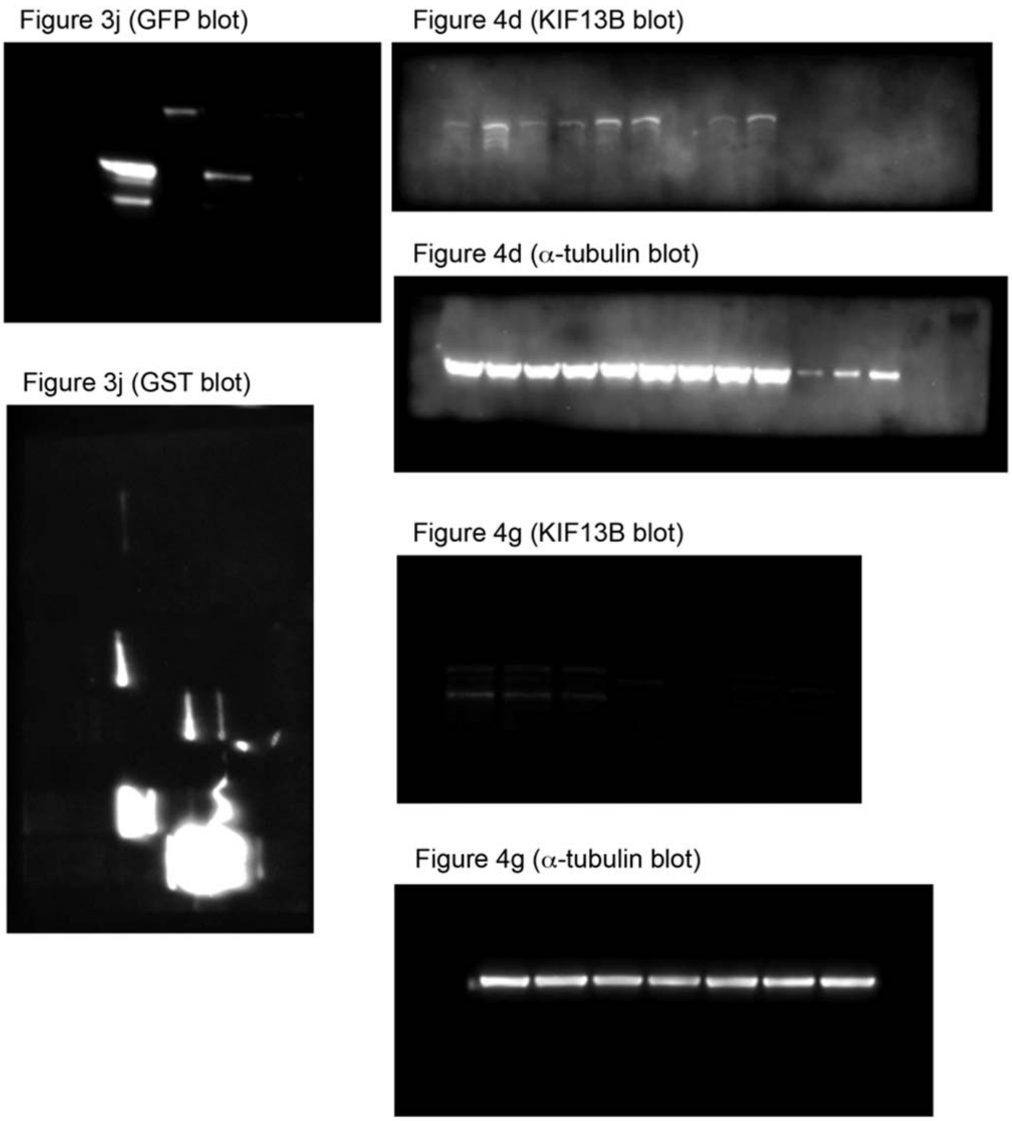

Supplementary Figure 10. Original blots for Figure 3j, Figure 4d and Figure 4g.

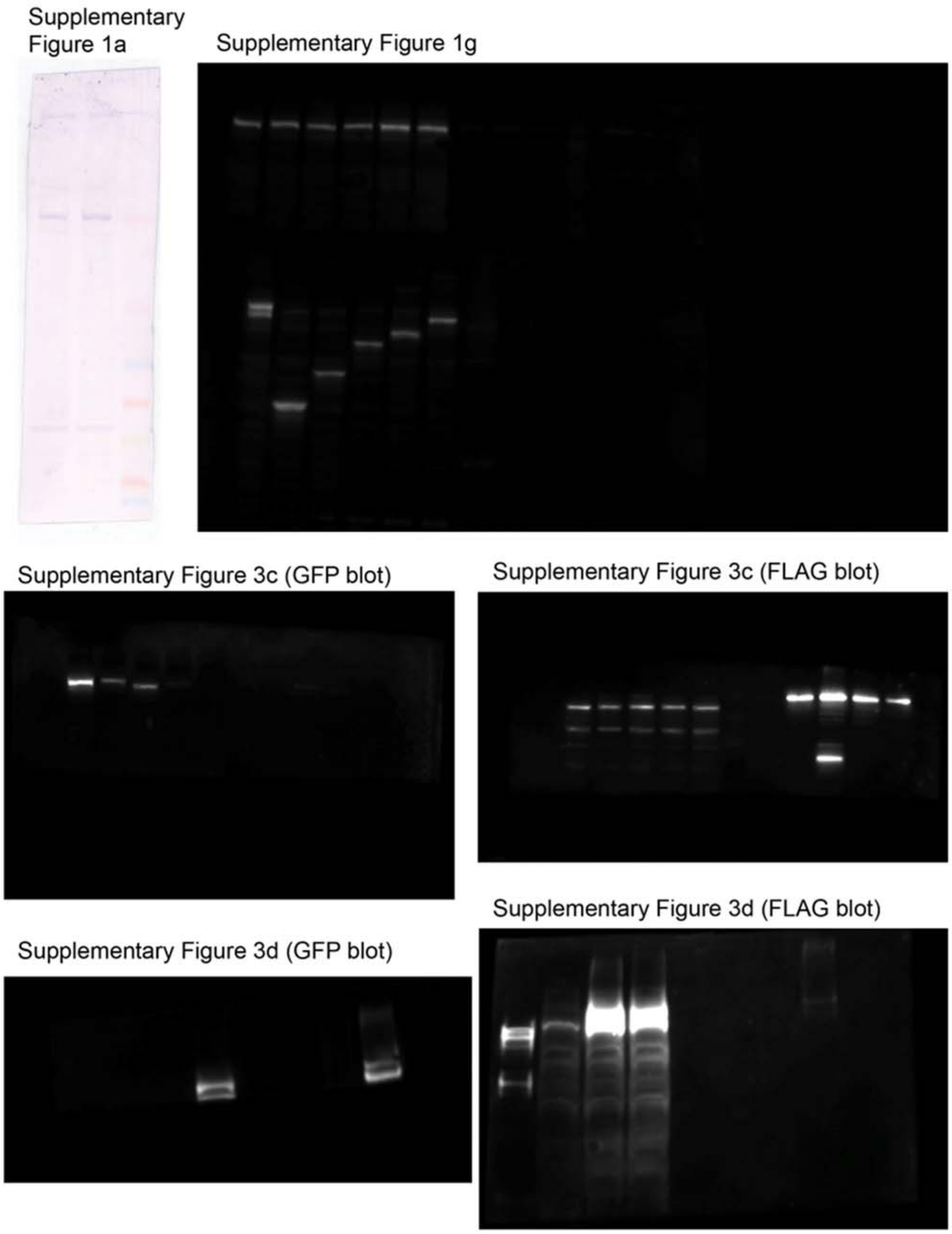

Supplementary Figure 11. Original blots for Supplementary Figures 1a, 1g, 3c and 3d.

Supplementary Figure 3f (GFP blot)

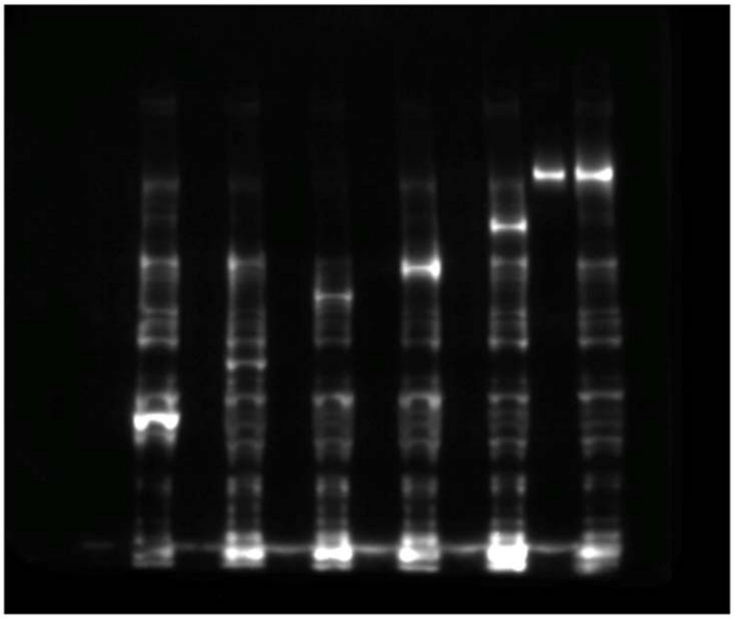

Supplementary Figure 3f (FLAG blot)

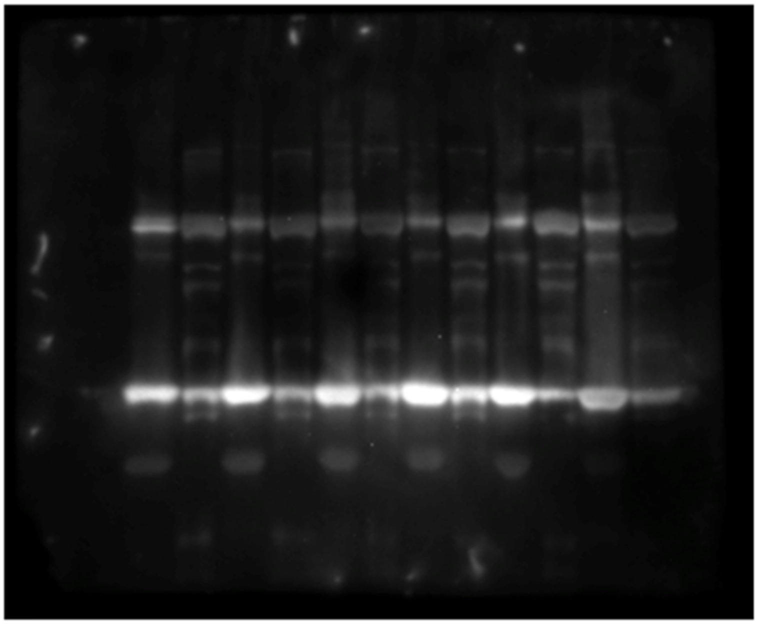

Supplementary Figure 12. Original blots for Supplementary Figure 3f.

Supplementary Table 1. Primers used in this study.

Primer name	Sequence 5'-3'*	Used for:
HsSP1	CTGTGCCCATCATGGTATAAG	*KIF13B* 5'RACE NT2 cells
HsSP2	CAGAGCCAGTCTGTCCATAGG	*KIF13B* 5'RACE NT2 cells
HsSP3	TTGACCTGCATACTTTTCTTTGAC	*KIF13B* 5'RACE NT2 cells
MmSP1	CTTTGGCTGGCCCCGGG	*Kif13b* 5'RACE MEF cells
MmSP2	CTTCTCTCGGACAGATTCATCC	*Kif13b* 5'RACE MEF cells
MmSP3	GGCACTTGAAAACATCTTCTTGACC	*Kif13b* 5'RACE MEF cells
Mm232bp	GCCGCTAGCGGAGAGGGAGGTACAGGAACAGC	*Kif13b* Promoter cloning in pGL3
Mm494bp	GCCGCTAGCCCATGGCCAGTTGGCTTTCTCTTTC	*Kif13b* promoter cloning in pGL3
Mm930bp	GCCGCTAGCCAGGGCCATTGAGATGGTTCAGTG	*Kif13b* promoter cloning in pGL3
MmPromluc495F	GCCGCTAGCCCATGGCCAGTTGGCTTTCTCTTTC	*Kif13b* promoter cloning in pGL3
LucRev	CCAGGAACCAGGGCGTATCTCTTCATAGCC	*Kif13b* promoter cloning in pGL3
MutCC1F	CTTTTCCCCCACAGC**AA**CCGCCCGCC	*Kif13b* EGR1 mutant promoter construct
MutCC1R	GGCGGGCGG**TT**GCTGTGGGGGAAAAG	*Kif13b* EGR1 mutant promoter construct
MutCC2F	CCCACAGCCCCCGC**AA**GCCGGGTT	*Kif13b* SP1 mutant promoter construct
MutCC2R	AACCCGGC**TT**GCGGGGGCTGTGGG	*Kif13b* SP1 mutant promoter construct
MutCC3F	GAGAGTTTGTCCGGTGG**AA**GAGGTAGACGG	*Kif13b* GC-box mutant promoter construct
MutCC3R	CCGTCTACCTC**TT**CCACCGGACAAACTCTC	*Kif13b* GC-box mutant promoter construct
KIF13B_1F	AAAAAGGTACCATGGGGGACTCCAAA GTG	GFP-KIF13B 1-370/860/1000/1289 fusions
KIF13B_1289R	AAAAATCTAGACATCTTTTTAGGAGAC	GFP-KIF13B 1-1289 fusion
KIF13B_1000R	AAAAATCTAGACTACTCATTCTGTTCCAAG	GFP-KIF13B 1-1000 fusion
KIF13B_860R	AAAAA TCTAGA CTA CTC AAA GGA GAC CTC TGC	GFP-KIF13B 1-860 fusion
13B_motorF	CCAAGCTT ATGGGGGACTCCAAAGTGAAG	GFP-KIF13B 1-557 fusion

13B_motorR	ATGGTACCGT GTCCTCATCCTCTCGTTCTG	GFP-KIF13B 1-557 fusion
KIF13B_370R	AAAAATCTAGACTATCCCGGATAATTCGGGC	GFP-KIF13B 1-370 fusion
KIF13B_561F	CCGGTACCTCCATGAAGAACGAGAATAGTTC	GFP-KIF13B 561-1826 fusion
KIF13B_1826R	AAGGATCCTCAGCTGGCCCAGGATTTC	GFP-KIF13B 561/840/1000/1650/ 1289-1826 fusions
KIF13B_840F	AAGGTACCCTCAGTGGTGATGTTGGGG	GFP-KIF13B 840-1826 fusion
KIF13B_1000F	GAGGTACCGAGAATGGTGAATACTGCCCT	GFP-KIF13B 1000-1826 fusion
KIF13B_1650F	AAGGTACCAGGGTGCGGGCCTCG	GFP-KIF13B 1650-1826 fusion
KIF13B_1289F	GCGGTACCATGTCTCATCGAAGTTCTATTC	GFP-KIF13B 1289-1702/1826 fusion
KIF13B_1702R	AAGGATCCTCATCGGAGCCACTCCGG	GFP-KIF13B 1289-1702 fusion
KIF13B_861F	AAAAGCTTTGAAGGAGACCCAGGAGAAC	GFP-KIF13B 861-1000 fusion
KIF13B_1000R	GAGGATCCCTCATTCTGTTCCAAGATTTGA	GFP-KIF13B 861-1000 fusion
NPHP4_1F	AAAAAGCGGCCGCAATGAACGACTGGCACAG G	FLAG-NPHP4 1-650/836/1426 fusions
NPHP4_1826R	AAAAAGATATCTCACTGGTAGATGACCTTCAC	FLAG-NPHP4 1/649/841-1426 fusions
NPHP4_836R	AAAAAGATATCCTACAACCTCTCACTTTC	FLAG-NPHP4 1-836 fusion
NPHP4_650R	AAAAAGATATCGGCAAGAAACTGTAGCAC	FLAG-NPHP4 1-650 fusion
NPHP4_841F	AAAAAGCGGCCGCAACATTGCCACCGTCCAG	FLAG-NPHP4 841-1426 fusion
NPHP4_649F	AAAAAGCGGCCGCAGCCTTTAGCAGAGTGGC C	FLAG-NPHP4 649-1426 fusion
NPHP4_C2F	AAGGATCCTTTAGCAGAGTGGCCCAG	GST-NPHP4 650-839 fusion
NPHP4_C2R	CCGAATTCACAACCTCTCACTTTCTGTTC	GST-NPHP4 650-839 fusion
HsWNT5A_F	CAAGGGCTCCGAGAGTG	*WNT5A* qPCR, RPE1 cells
HsWNT5A_R	ATCTCTCCTTCAGGGCATCA	*WNT5A* qPCR, RPE1 cells
HsGLI1_F	CAGGGAGTGCAGCCAATACAG	*GLI1* qPCR, RPE1 cells
HsGLI1_R	GAGCGGCGGCTGACAGTATA	*GLI1* qPCR, RPE1 cells

HsB2M_F	GTTTACTCACGTCATCCAGCAGAGAATG	Reference gene for qPCR, RPE1 cells
HsB2M_R	CCATGTTTGATGTATCTGAGCAGGTTGC	Reference gene for qPCR, RPE1 cells
13B_F1_260916	TAAGTTGTAGCAAACGACTCGC	Sequencing of *KIF13B*$^{-/-}$ RPE1 cells
13B_R1_260916	CGGGGGCGACGATGAC	Sequencing of *KIF13B*$^{-/-}$ RPE1 cells

*For primers used for mutant promoter constructs, the nucleotides that are changed relative to the *Kif13b* promoter sequence are in bold. For some primers, relevant restriction endonuclease sites are included (underlined).

Supplementary references

1. Kuhn R. M., Haussler D., Kent W. J. The UCSC genome browser and associated tools. *Briefings in bioinformatics*, **14**, 144-161 (2013).
